# Chorea-related mutations in PDE10A result in aberrant compartmentalization and functionality of the enzyme

**DOI:** 10.1073/pnas.1916398117

**Published:** 2019-12-23

**Authors:** Gonzalo S. Tejeda, Ellanor L. Whiteley, Tarek Z. Deeb, Roland W. Bürli, Stephen J. Moss, Eamonn Sheridan, Nicholas J. Brandon, George S. Baillie

**Affiliations:** ^a^Institute of Cardiovascular and Medical Science, University of Glasgow, G12 8QQ Glasgow, United Kingdom;; ^b^AstraZeneca-Tufts University Laboratory for Basic and Translational Neuroscience Research, Tufts University School of Medicine, Boston, MA 02111;; ^c^Neuroscience, IMED Biotech Unit, AstraZeneca, CB21 6GH Cambridge, United Kingdom;; ^d^Department of Neuroscience, Tufts University School of Medicine, Boston, MA 02111;; ^e^Institute of Biomedical and Clinical Sciences, University of Leeds, LS9 7TF Leeds, United Kingdom;; ^f^Neuroscience, IMED Biotech Unit, AstraZeneca, Boston, MA 024515

**Keywords:** PDE10A, Huntington’s disease, cyclic AMP, GAF domain, phosphodieaterase

## Abstract

A robust body of evidence supports the concept that phosphodiesterase 10A (PDE10A) activity in the basal ganglia orchestrates the control of coordinated movement in human subjects. Although human mutations in the PDE10A gene manifest in hyperkinetic movement disorders that phenocopy many features of early Huntington’s disease, characterization of the maladapted molecular mechanisms and aberrant signaling processes that underpin these conditions remains scarce. Recessive mutations in the GAF-A domain have been shown to impair PDE10A function due to the loss of striatal PDE10A protein levels, but here we show that this paucity is caused by irregular intracellular trafficking and increased PDE10A degradation in the cytosolic compartment. In contrast to GAF-A mutants, dominant mutations in the GAF-B domain of PDE10A induce PDE10A misfolding, a common pathological phenotype in many neurodegenerative diseases. These data demonstrate that the function of striatal PDE10A is compromised in disorders where disease-associated mutations trigger a reduction in the fidelity of PDE compartmentalization.

## Significance

Phosphodiesterase 10A (PDE10A) is as a target of interest in Huntington’s disease (HD) as levels of the enzyme have been shown to decrease prior to the development of the hallmark motor symptoms. Clearly, a better understanding of how PDE10A protein levels change as HD develops is required. Here we show that mutations in the regulatory GAF domains of PDE10A that cause hyperkinetic syndromes in humans lead to misprocessing of the PDE10A enzyme that ultimately leads to targeted degradation by the ubiquitin proteasome system or clearance by autophagy. Both mechanisms result in a paucity of PDE10A activity that lead to a loss of movement coordination. Our research suggests that similar mechanisms may underpin PDE10A loss during HD.

Cyclic nucleotide signaling plays a critical role in the regulation of coordinated movement in the basal ganglia. The duration, amplitude, and subcellular localization of cyclic adenosine monophosphate (cAMP) and cyclic guanosine monophosphate (cGMP) signals are tightly regulated by phosphodiesterases (PDEs). PDE10A coordinates cAMP signaling in striatal medium spiny neurons, the main input region of the basal ganglia circuitry and the most vulnerable cells to degeneration during Huntington’s disease (HD) (1, 2). This distribution pattern positions PDE10A as an effector in motor control and as an attractive therapeutic target for basal ganglia diseases. Furthermore, PDE10A protein levels are reduced in HD and Parkinson’s disease (3–5), with the decline preceding the onset of motor symptoms and progressing over the course of the disease (6–12). The role of PDE10A in the regulation of coordinated movement has been further established by both dominant and recessive mutations in this gene as causes of childhood-onset hyperkinetic syndromes (13–16). Patients with dominant heterozygous mutations in PDE10A (F300L, F334L, and F334C) displayed symmetrical striatal bilateral lesions, as shown by MRI (14–17). These substitutions located in the regulatory GAF-B domain do not alter the basal activity of PDE10A but they inhibit the cAMP-stimulatory effect for cGMP hydrolysis, as reported for F300L and F334L mutations (15). Moreover, subjects carrying the F300L modification showed a significant decrease in striatal PDE10A levels (17). In contrast, familial homozygous mutations in PDE10A have been detected in a conserved region of the GAF-A domain (Y107C and A116P) and MRI imaging of affected individuals did not show alterations in overall brain structure (13). The motor abnormalities produced by the GAF-A mutations are the result of reduced PDE10A activity in the striatum due to a loss in protein levels.

It is expected that the disease phenotypes are due to the aberrant function of the major striatal splice variant PDE10A2. This isoform is located in the plasma membrane associated with other synaptic proteins (18). Translocation and anchoring of PDE10A2 to the membrane is achieved by irreversible palmitoylation at C11 of the newly synthesized enzyme, although this posttranslational modification can be prevented by phosphorylation at T16 by protein kinase A (PKA) (19). The 2 other prevalent isoforms in humans, PDE10A1 and PDE10A19, have a cytosolic localization due to the lack of the N-terminal cysteine residue (20, 21). In transgenic HD models, the loss of striatal PDE10A2 is thought to be the consequence of transcriptional repression by mutant huntingtin (mHTT) (3). However, protein degradation could also play a role in the down-regulation of PDE10A in these diseases. Protein stability of this enzyme seems to be regulated by the ubiquitin proteasome system (UPS), as the increased expression of proteasomal components reduces PDE10A levels in striatal neurons of an HD mouse model (22). Additionally, activation of lysosomal function by overexpression of the transcription factor EB decreases PDE10A levels in the same HD model (23), suggesting an alternative route of degradation that could be isoform-specific or dependent on differential subcellular localization (24). This complexity hinders the search for the effectors involved in PDE10A dysregulation, while the pathological mutations in this gene offer an opportunity for a straightforward identification of these mechanisms. In this work we compare the basic cellular biology and proteostasis of the GAF-A and GAF-B PDE10A mutants and propose molecular mechanisms responsible for reduced PDE10A levels in affected individuals.

## Results

### Pathological Mutations in the GAF-A Domain of PDE10A Impair Its Enzymatic Activity at the Plasma Membrane.

**Fig. 1.**
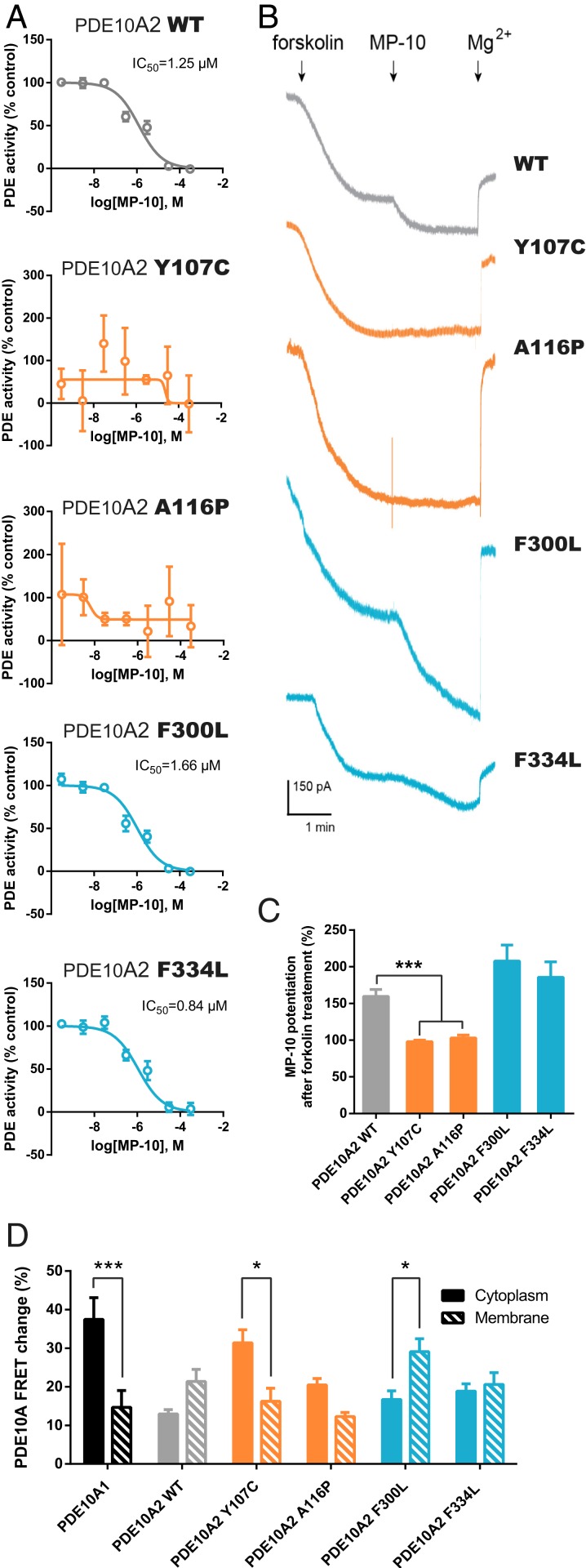
Chorea-related mutations in GAF-A but not GAF-B domains of PDE10A alter enzymatic activity. (*A*) Dose-dependent inhibition of PDE10A activity in membrane fractions of HEK293 cells transfected with the different forms of PDE10A2 as shown, over a range of 0.3 μM to 3 mM MP10 and using 2 μM cAMP as substrate. Inhibition profiles represent the mean ± SEM of the percentage of PDE activity relative to a control without MP10 treatment. (*B*) Representative traces of evoked CNG-mediated currents recorded from HEK293 cells expressing the different PDE10A forms. The time of application of 5 μM forskolin, 300 nM MP10, and 10 mM MgCl_2_ is indicated. (*C*) Quantification of the percentage increase of CNG-mediated potentiation by MP-10 treatment, relative to the forskolin response. Data are represented as the mean ± SEM and results analyzed by one-way ANOVA test followed by Dunnett’s post hoc (***P = 0.0008; *n* = 5). (*D*) Quantification of the FRET change in the cytosolic and membrane compartments in HEK293 cells transfected with PDE10A1 or the different forms of PDE10A2. Results are represented as mean ± SEM of the FRET response obtained with 300 nM MP-10 treatment after subtracting the endogenous signal recorded with a previous application of 5 μM forskolin and relative to the saturating response (25 μM forskolin and 100 μM IBMX). Differences were evaluated by a two-way ANOVA followed by Bonferroni’s post hoc (*P = 0.0453 for Y107C and 0.0405 for F300L, ***P < 0.0001; *n* = 15).

Disease phenotypes caused by PDE10A GAF-A and GAF-B mutations have been linked to aberrant PDE activity. Thus, in vitro PDE assays have shown a diminished capacity to degrade cAMP in striatal lysates from a knockin mouse carrying the mutated PDE10A GAF-A variant homologous to the human Y107C (13). Furthermore, experiments in membrane fractions of COS7 cells with the F300L and F334L PDE10A variants exhibited a normal enzymatic basal activity but an impaired cAMP-stimulatory effect for cGMP hydrolysis (15). Considering that both PDE10A Y107C and F300L mutants share pathological features, we first wanted to compare their enzymatic activities. As phenotypes underpinned by PDE10A mutations are caused by functional abnormality of the most abundant isoform, we generated plasmids containing PDE10A2-FLAG with the human GAF-A mutations Y107C and A116P and the GAF-B mutations F300L and F334L. Membrane fractions from HEK293 cells transiently transfected with these constructs or the PDE10A2 WT were used for in vitro assays to measure PDE activity (Fig. 1*A*). The ability to hydrolyze cAMP with increasing concentrations of the highly selective PDE10A inhibitor MP10 was similar between PDE10A2 WT, F300L, and F334L forms, confirming previous reports (15). In contrast, PDE activity of the Y107C or the A116P mutants was not measurable in the membrane fraction, consistent with the results from the knockin mouse (13). However, these assays may not reflect the cAMP dynamics in the membrane of a living cell.

In light of this, we performed electrophysiological experiments to monitor changes in intracellular cAMP levels due to the activity of these PDE10A mutants. We overexpressed cyclic nucleotide-gated (CNG) channels in HEK293 cells as a cAMP biosensor (25, 26), ADCY3 to stimulate endogenous cAMP production, one of the PDE10A2 variants, and GFP. Cultures were preincubated with rolipram until a stable baseline was achieved, in order to eliminate endogenous PDE4 activity that can prevent CNG channel activation (27). Then, cells were treated with forskolin and PDE10A activity was assessed by measuring the increase in current after subsequent addition of MP10 (Fig. 1*B*). Cells expressing PDE10A2 WT and GAF-B mutant forms exhibited an increase in the CNG-mediated currents after MP10 treatment (Fig. 1*C*). In stark contrast, MP10 application did not further activate CNG currents in cells expressing the Y107C or A116P variants. Do the mutations in the GAF-A domain abolish the capacity of PDE10A2 to hydrolyze cAMP? Or do they affect its trafficking to the plasma membrane as depending on palmitoylation levels (19)? To explore these possibilities, we compared cAMP fluctuations in the cytosol and the plasma membrane using a FRET reporter. Cytosolic or membrane cAMP FRET sensors alongside the different PDE10A2 variants or the cytosolic isoform PDE10A1 were coexpressed in HEK293 cells (*SI Appendix*, Fig. S1*A*) (28). Cultures were treated with forskolin, followed by MP10 to assess PDE10A activity. As expected, cells with PDE10A1 exhibited a dramatic increase in FRET response in the cytosol after treatment with MP10, which was absent from the membrane (Fig. 1*D*). In contrast, overexpression of PDE10A2 F300L caused a higher cAMP change in the plasma membrane, resembling the response obtained with the WT. Interestingly, cells expressing the PDE10A2 GAF-A mutants displayed a marked FRET change in the cytosol. This suggests that the pathological mutations in the GAF-A domain of PDE10A2 do not hinder enzymatic activity per se, but reduce the translocation of the protein to the plasma membrane.

### Mutations in GAF-A and GAF-B Domains of PDE10A Differentially Alter the Subcellular Localization of PDE10A Protein.

**Fig. 2.**
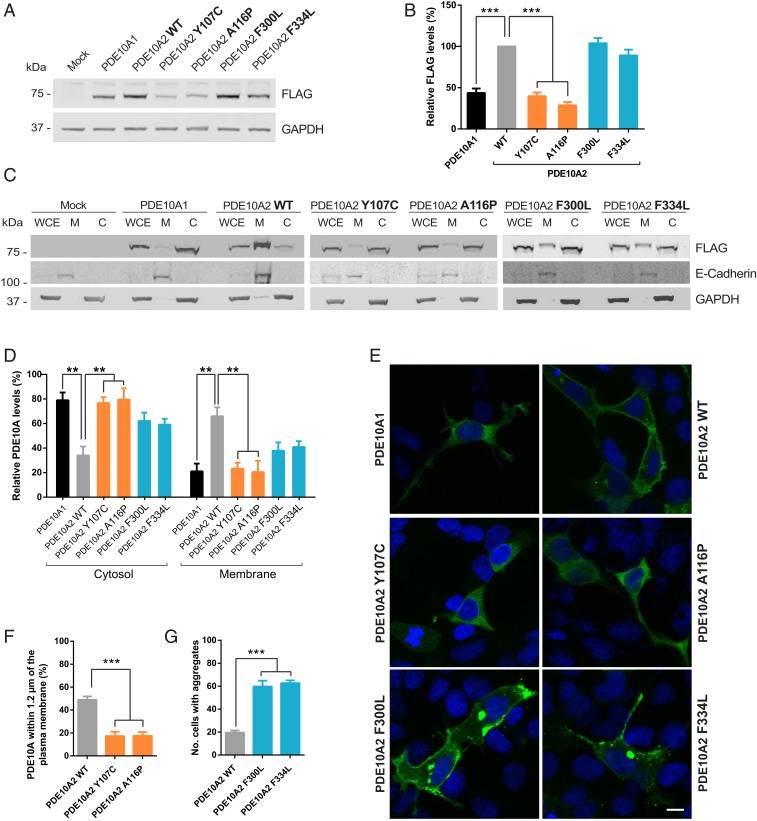
Mutations in the GAF domains of PDE10A induce abnormal cellular compartmentalization. (*A*) Comparison of protein levels of recombinant PDE10A1 or the different forms of PDE10A2 tagged with FLAG expressed in HEK293 cells compared to a mock condition. (*B*) Quantification of PDE10A in transfected HEK293 cells normalized to GAPDH levels. Data are presented as mean ± SEM relative to values obtained for PDE10A2 WT and statistical analysis was performed by one-way ANOVA test followed by post hoc Tukey test (***P < 0.0001; *n* = 8). (*C*) Subcellular fractionation of the PDE10A-FLAG variants in transfected HEK293 cells showing the membrane (M) and cytosolic (C) compartments compared to whole-cell extracts (WCE). (*D*) Densitometric analysis of PDE10A levels in the membrane and cytosolic fractions, normalized to whole-cell homogenate. Results are represented as mean ± SEM and significance was calculated by a two-way ANOVA test followed by Tukey’s post hoc (**P = 0.0012 for PDE10A1, 0.0011 for Y107C, and 0.0021 for A116P; *n* = 3). (*E*) Analysis of the recombinant PDE10A-FLAG forms distribution in transfected HEK293 cells (FLAG in green and DAPI in blue). (Scale bar, 10 μm.) (*F*) Analysis of PDE10A2 signal within 1.2 μm of the plasma membrane in GAF-A mutants compared to the WT form. Results are presented as mean ± SEM and significance was calculated by one-way ANOVA followed by Dunnett’s post hoc (***P < 0.0001; *n* = 6). (*G*) Quantification of the percentage of HEK293 cells containing PDE10A2 aggregates when overexpressing the WT or the GAF-B mutants forms. The graph depicts the mean ± SEM and the statistical analysis was performed by one-way ANOVA followed by Dunnett’s post hoc (***P < 0.0001; *n* = 19).

The depleted activity of PDE10A2 Y107C and A116P observed at the plasma membrane compelled us to evaluate the expression and localization of the GAF-A and GAF-B mutants. Initially, we compared the transfected GAF-A and GAF-B mutations levels to WT forms. Analysis of mRNA levels showed no difference in expression among all PDE10A variants (*SI Appendix*, Fig. S1*B*), but immunoblots detecting the recombinant proteins (Fig. 2*A*) confirmed significantly lower levels of Y107C and A116P mutants in comparison to PDE10A2 WT (Fig. 2*B*), similar to PDE10A1. Next, we studied cellular distribution. Crude fractionation confirmed that PDE10A1 WT was primarily in the cytosol, whereas PDE10A2 WT was mainly located in the plasma membrane (Fig. 2*C*). However, a fraction of PDE10A2 could also be detected in the cytosol (Fig. 2*D*), which might be attributed to the PKA-phosphorylated pool (19). Interestingly, both PDE10A2 Y107C and A116P were observed at significantly greater levels in the cytosolic fraction than the WT form. The PDE10A2 GAF-B mutants also showed a substantial reduction in the membrane-to-cytosol ratio, although proportion in the membrane was higher than in the GAF-A mutants. Validation of the cytosolic and membrane fractions was confirmed by probing for GAPDH and E-cadherin, respectively (Fig. 2*C*).

To extend these findings, we performed immunofluorescence studies to visualize the distribution of PDE10A forms in transfected HEK293 (Fig. 2*E*), SH-SY5Y (*SI Appendix*, Fig. S1*C*), and PC12 cells (*SI Appendix*, Fig. S1*D*). Confocal images corroborated the cytosolic localization of PDE10A1 and the predominantly membrane staining of PDE10A2 WT. Consistent with the fractionation studies, mutations in the GAF-A domain of PDE10A2 abolished plasma membrane localization and showed a cytosolic distribution (Fig. 2*F*). Interestingly, both PDE10A2 GAF-B mutants extensively accumulated in aggregates within the cytoplasm and the perinuclear region in ∼75% of transfected cells (Fig. 2*G*). However, a reduced proportion of these forms could still be observed at the plasma membrane. Smaller aggregates were also detected in cells with PDE10A2 WT, probably due to overexpression, but significantly lower than that observed for F300L and F334L mutants. These results suggest that GAF-A and GAF-B mutants alter PDE10A distribution in different ways, likely dysregulating cyclic nucleotide levels within different compartments, which might be important to explain the differences in clinical presentation. Furthermore, changes in cAMP levels induced by inhibition of endogenous PDE4 do not seem to affect the distribution of the different PDE10A2 variants (*SI Appendix*, Fig. S1*E*).

### Mechanism of PDE10A Degradation Depends on Its Subcellular Localization.

**Fig. 3.**
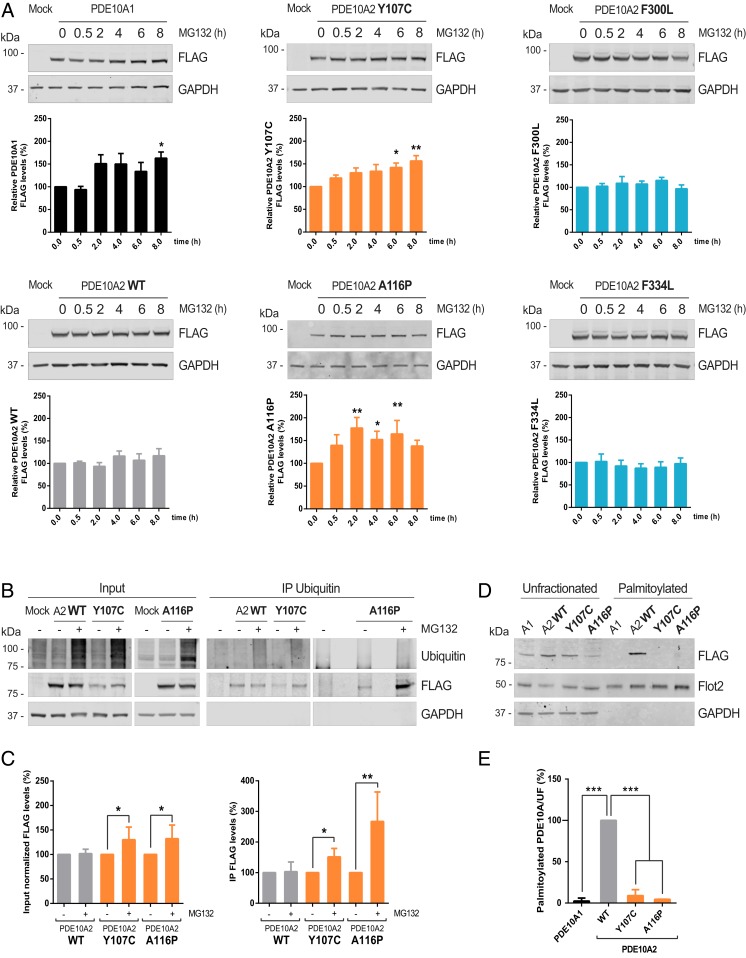
Mutations in the PDE10A2 GAF-A domain impair translocation to the plasma membrane and increase their turnover. (*A*) Time courses of PDE10A degradation by UPS. HEK293 cells with PDE10A1 or the different variants of PDE10A2 were treated with MG132 (20 µM). Graphs below immunoblots represent mean ± SEM of PDE10A levels normalized to GAPDH and relative to the control without treatment. Significance was calculated by one-way ANOVA followed by Dunnett’s post hoc test (*P = 0.0482 for PDE10A1, 0.0419 for Y107C, and 0.0490 for A116P; **P = 0.0029 for Y107C, 0.0010 and 0.0091 for 2- and 6-h A116P, respectively; *n* = 4 to 7). (*B*) Ubiquitin levels of PDE10A2 WT and Y107C or A116P forms after UPS inhibition. The presence of PDE10A-FLAG was evaluated in immunoprecipitates (IP) of ubiquitinated proteins from lysates of transfected HEK293 cells treated with MG132 (20 µM) for 5 h. (*C*) Quantification of PDE10A levels in the input (*Left*, normalized with GAPDH) or immunoprecipitated fractions (*Right*) relative to untreated cells expressing the same recombinant proteins. Values are presented as mean ± SEM and statistical analysis was performed by one-way ANOVA followed by Bonferroni post hoc test (*P = 0. 0.0266 for input Y107C, 0.0411 for A116P, and 0.0278 for IP Y107C; **P = 0.0011; *n* = 3). (*D*) Palmitoylated proteins were isolated from HEK293 cells expressing PDE10A1, PDE10A2 WT, Y107C, or A116P and compared to the unfractionated samples. (*E*) Quantification of palmitoylated PDE10A levels expressed as the mean ± SEM of the palmitoylated/unfractionated ratio relative to PDE10A2 WT. Significance was analyzed by one-way ANOVA followed by Dunnett’s post hoc test (***P < 0.0001 for PDE10A1 and A116P and 0.0002 for Y107C; *n* = 3).

It has been suggested that proteostasis depends on subcellular localization, with cytosolic proteins being subjected to UPS degradation, while membrane proteins usually undergo proteolysis through the autophagy-lysosomal pathway (ALP) (29). Consequently, we investigated if the subcellular mislocalization of PDE10A mutants changed the route of PDE10A proteostasis. To test this hypothesis, transfected cells were treated with MG132 for various periods of time (Fig. 3*A*). PDE10A1 significantly accumulated after proteasome inhibition while the membrane-anchored PDE10A2 WT levels did not. Similarly, protein levels of both GAF-B mutations remained stable throughout the time course. A different result was obtained for the GAF-A mutants where significantly elevated protein levels were observed after MG132 treatment. These results indicate that the subcellular localization of PDE10A is critical for the action of the UPS. However, this apparent increased degradation of PDE10A2 Y107C and A116P could also be a consequence of aberrant ubiquitination. Therefore, we decided to immunoprecipitate ubiquitinated proteins following proteasomal inhibition (Fig. 3*B*). Immunoblotting for the FLAG tag showed that both PDE10A2 WT and the GAF-A mutant forms can be ubiquitinated. Moreover, MG132 treatment significantly increased the levels of the coimmunoprecipitated Y107C and A116P variants (Fig. 3*C*), indicating that GAF-A forms are more susceptible to ubiquitin-mediated protein degradation. Next, we wanted to evaluate if the GAF-A mutations impair the recognition and palmitoylation of the enzyme. We purified palmitoylated proteins using resin-assisted capture of acylated proteins from cells expressing PDE10A1 or the different forms of PDE10A2. The highly palmitoylated protein flotillin-2 (30) was used to validate isolation of the palmitoylated fraction. PDE10A1 did not exhibit any palmitoylation, in contrast to the robustly palmitoylated PDE10A2 WT (Fig. 3*D*), as previously described (19). Accordingly, Y107C and A116P mutations significantly abolished the palmitoylation of PDE10A2 (Fig. 3*E*). Conversely, mutations in the GAF-B domain did not affect palmitatoylation of PDE10A2 in a significant manner (*SI Appendix*, Fig. S2 *A* and *B*). Collectively, these results indicate that chorea-related mutations in the GAF-A domain of PDE10A2 impair its palmitoylation and trafficking to the plasma membrane. This causes accumulation in the cytosol, which increases its likelihood of being targeted by the UPS. This concept could help explain the reduction in striatal PDE10A observed in affected individuals (13).

**Fig. 4.**
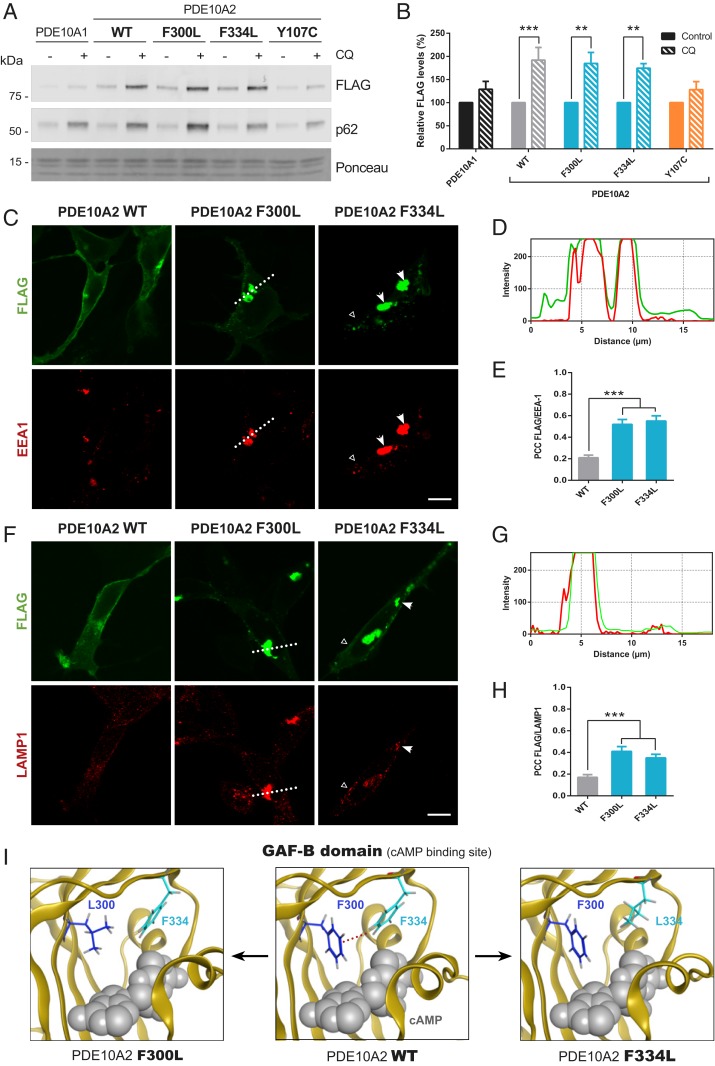
Aggregates of PDE10A, induced by the loss of aromatic–aromatic interactions in GAF-B mutants, are cleared by autophagy. (*A*) Analysis of PDE10A degradation by the lysosome. HEK293 cells expressing PDE10A1 or PDE10A2 variants were treated with chloroquine (CQ, 10 µM) over 12 h and the Triton X-100 insoluble fraction was evaluated. (*B*) Quantification of PDE10A levels in the insoluble fraction after CQ treatment, normalized to stable bands in Ponceau S staining. Data are presented as mean ± SEM relative to values obtained for the nontreated samples and statistical analysis was performed by two-way ANOVA test followed by Bonferroni’s post hoc test (**P = 0.0010 for F300L and 0.0040 for F334L; ***P = 0.0004; *n* = 4). (*C* and *F*) Analysis of the colocalization between PDE10A2-FLAG forms (green) and EEA1 (*C*) or LAMP1 (*F*, red) in transfected HEK293 cells. Arrows indicate presence of EEA1 or LAMP1 in PDE10A aggregates although lack of colocalization could also be observed (empty arrowheads). (Scale bars, 10 μm.) (*D* and *G*) Line scan intensity profiles corresponding to the section shown in the PDE10A2 F300L condition (dotted line). (*E* and *H*) Quantification of colocalization of EEA1 (*E*) or LAMP1 (*H*) with the indicated PDE10A2 variant using PCC. Statistical analysis was calculated with one-way ANOVA followed by Dunnett’s post hoc test (***P < 0.0001 in *E*, *n* = 18; and ***P < 0.0001 for F300L and 0.0006 for F334L in *H*, *n* = 17). (*I*) Visualization of the cAMP binding site within the GAF-B domain of PDE10A. (*Left*) GAF-B domain containing the F300L mutation (conformation minimized using MOE). (*Center*) WT GAF-B; red dotted line indicates the edge-to-face interaction between F300 and F334. (*Right*) GAF-B domain containing the F334L mutation.

The resistance of PDE10A2 WT, F300L, and F334L forms to UPS degradation suggests an alternative route, possibly by the ALP. In fact, both membrane proteins and insoluble aggregates are normally eliminated through autophagy (29, 31), a process that can be controlled by ubiquitination (32). Consequently, we studied the effect of inhibiting lysosomal degradation on PDE10A2 forms present in the soluble (*SI Appendix*, Fig. S2*C*) and insoluble fractions (Fig. 4*A*). Under these conditions, the levels of all recombinant PDE10A2 variants tested remained unchanged in the soluble fraction (*SI Appendix*, Fig. S2*D*), but a significant increase of PDE10A2 WT, F300L, and F334L forms was evident in the insoluble fraction (Fig. 4*B*). ALP inhibition was confirmed by the accumulation of the autophagosome marker p62 (33), which was enriched in the insoluble fraction. The small proportion of the cytosolic forms in the triton-insoluble fraction could be explained by their interaction with components of the detergent-insoluble cytoskeleton, as it has been shown that PDE10A can bind to cytoskeleton-interacting AKAP150 in striatal neurons (18, 34). In order to confirm that the aggregates of PDE10A2 GAF-B mutants are ultimately cleared by the autophagic system, we examined their colocalization with the endosomal marker EEA1 (Fig. 4*C*), which has a role in the fusion with autophagosomes (35), and the lysosomal marker LAMP1 (Fig. 4*F*). Line-scan intensity profiles across the perinuclear region in confocal images (Fig. 4*D*) showed that the aggregates were contained in EEA1^+^-endosomes (verified by quantitative analysis using Pearson’s correlation coefficient [PCC]), in contrast to the PDE10A2 WT form (Fig. 4*E*). Immunostaining also revealed that the inclusions moderately overlapped with the lysosome marker (Fig. 4 *G* and *H*), illustrating a connection with the ALP.

Aggregation of the PDE10A2 GAF-B mutants could be explained by changes in noncovalent forces between the affected conserved aromatic residues. The conformation of the WT GAF-B domain is likely stabilized by an edge-to-face interaction between F300 and F334 (Fig. 4 *I*, *Center*). Both mutated forms (F300L and F334L) lack this positive interaction and therefore may display increased conformational flexibility in this region of the β-sheet. Ultimately, this change in mobility could promote propensity for aggregation of the GAF-B forms carrying these mutations.

### PDE10A2 F300L and F334L Mutants Accumulate in Aggresomes.

**Fig. 5.**
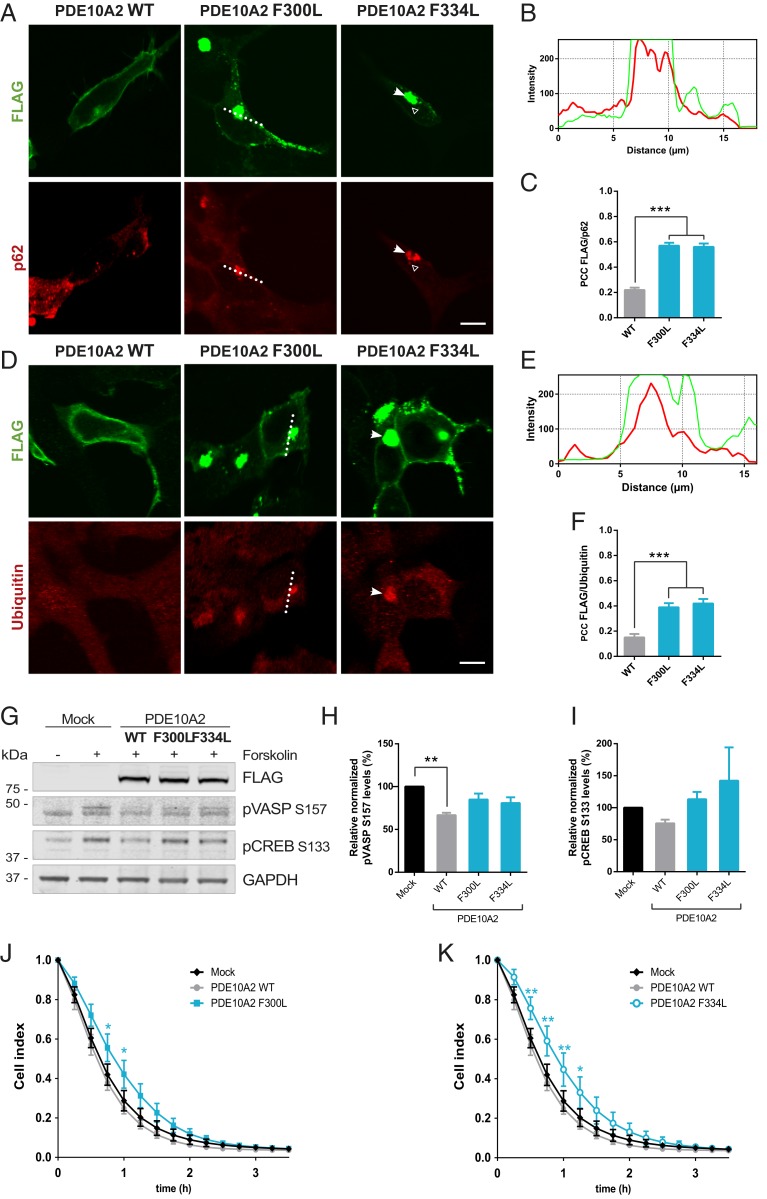
PDE10A GAF-B mutants accumulate in aggresomes. (*A* and *D*) Analysis of the colocalization between the PDE10A2-FLAG (green) forms and p62 (*A*) or ubiquitin (*D*, red). Arrows indicate recruitment of p62 and ubiquitin to PDE10A aggregates. (Scale bars, 10 μm.) (*B* and *E*) Surface line intensity profiles corresponding to the section shown in the cell expressing the PDE10A2 F300L form (dotted line). (*C* and *F*) Quantification of the PCCs between the indicated PDE10A2-FLAG forms and p62 (*C*) or ubiquitin (*F*). Significance was calculated with one-way ANOVA followed by Dunnett’s post hoc test (***P < 0.0001; *n* = 60 in *C* and 30 in *F*). (*G*) Analysis of PDE10A effect on forskolin-activated proteins. HEK293 cells expressing PDE10A WT, F300L, or F334L were treated with forskolin (25 µM) for 15 min and compared to a mock condition. (*H* and *I*) Quantification of phosphorylated VASP (in S157, *H*) or CREB (in S133, *I*) levels after forskolin treatment in the presence of the indicated PDE10A variants relative to mock and normalized to GAPDH. Graphs depict mean ± SEM and significance was calculated by one-way ANOVA followed by Dunnett’s post hoc test (**P = 0.0041; *n* = 3). (*J* and *K*) Cell survival analysis of transfected HEK293 cells with PDE10A2 F300L (*J*) or F334L (*K*) treated with 0.5 mM sodium arsenite compared to a mock condition and cells expressing the WT form. Data were recorded using the real-time electrical impedance‐based xCELLigence system and represented as mean ± SEM. Statistical analysis was performed by two-way ANOVA followed by Tukey’s post hoc test (*P < 0.05, **P < 0.01; *n* = 5).

Misfolded proteins such as PDE10A2 GAF-B mutants are resistant to proteasomal degradation and are processed by autophagy (31). Such aggregates are cleared into aggresomes, a pathological feature common in several neurodegenerative diseases (36). A hallmark of aggresomes is the enclosure by vimentin cages (37) and we could observe the redistribution of this intermediate filament around the PDE10A2 GAF-B mutant aggregates (*SI Appendix*, Fig. S2*A*). Furthermore, a known component of aggresomes, p62, was recruited into the PDE10A2 F300L and F334L inclusions (Fig. 5 *A* and *B*), increasing their colocalization 3-fold compared to the WT form (Fig. 5*C*). Considering that p62 often interacts with polyubiquitinated substrates in aggresomes (37), we also evaluated the ubiquitin levels of PDE10A2 mutant aggregates by immunofluorescence (Fig. 5*D*) and detected ubiquitin enrichment in large inclusions (Fig. 5 *E* and *F*). Furthermore, we also confirmed that these structures were not part of the Golgi or endoplasmic reticulum complexes (*SI Appendix*, Fig. S2*F*). Surprisingly, the modulator of aggresome formation HDAC6 (38) did not relocate to the GAF-B mutant inclusions (*SI Appendix*, Fig. S3*A*). Similarly, we also explored the possibility that these aggregates were derived from stress granules (SG) or P-bodies. We found a strong colocalization of TIA-1, critical for the early stages of SG-assembly, with the F300L and F334L aggregates (*SI Appendix*, Fig. S3*B*). However, treatment with the stress-inducer sodium arsenite did not increase costaining with TIA-1 and the mutant forms were not recruited into the newly formed SGs. Additionally, neither the SG marker G3BP nor the P-body component Dcp1a accumulated into the mutant PDE10A2 aggregates (*SI Appendix*, Fig. S3 *C* and *D*). Nevertheless, it has been reported that TIA-1 can integrate into insoluble inclusions generated by accumulation of mHTT fragments or misfolded tau (39), and many other proteins are also shared among the stress-response structures (40).

We decided to evaluate whether the sequestration PDE10A GAF-B mutations affected the PKA substrate target profile, such as vasodilator-stimulated protein (VASP) and cAMP response element-binding protein (CREB) (Fig. 5*G*) (13, 41). Phosphorylation of these substrates was significantly decreased for PDE10A2 WT but not with F300L or F334L forms (Fig. 5 *H* and *I*), showing that these mutations can modify the overall cAMP signaling dynamics in the cell. As protein inclusions or their intermediate species have repeatedly been associated with the cytotoxicity that underlies cell death in many neurodegenerative diseases (42, 43), we next evaluated the effect of PDE10A aggregates on cellular survival using the xCELLigence system. No significant differences in cell growth were recorded in cells overexpressing the GAF-B mutants compared to the WT form (*SI Appendix*, Fig. S3*E*). Thus, we investigated the survival response of these cells under sodium arsenite treatment. Surprisingly, expression of the aggregate-prone F300L and F334L mutants demonstrated a higher resistance to stress-induced death than cells transfected with the WT variant or a mock control (Fig. 5 *J* and *K*). This slower death rate could be consequence of a dysfunctional ALP that would prevent an excessive elevation of autophagy after high doses of sodium arsenite. For example, inhibition of the autophagic pathway by taurine treatment in HepG2 cells protects against arsenite-induced toxicity (44, 45).

### PDE10A2 Mutants Exhibit Differential Compartmentalization in Striatal Neurons.

**Fig. 6.**
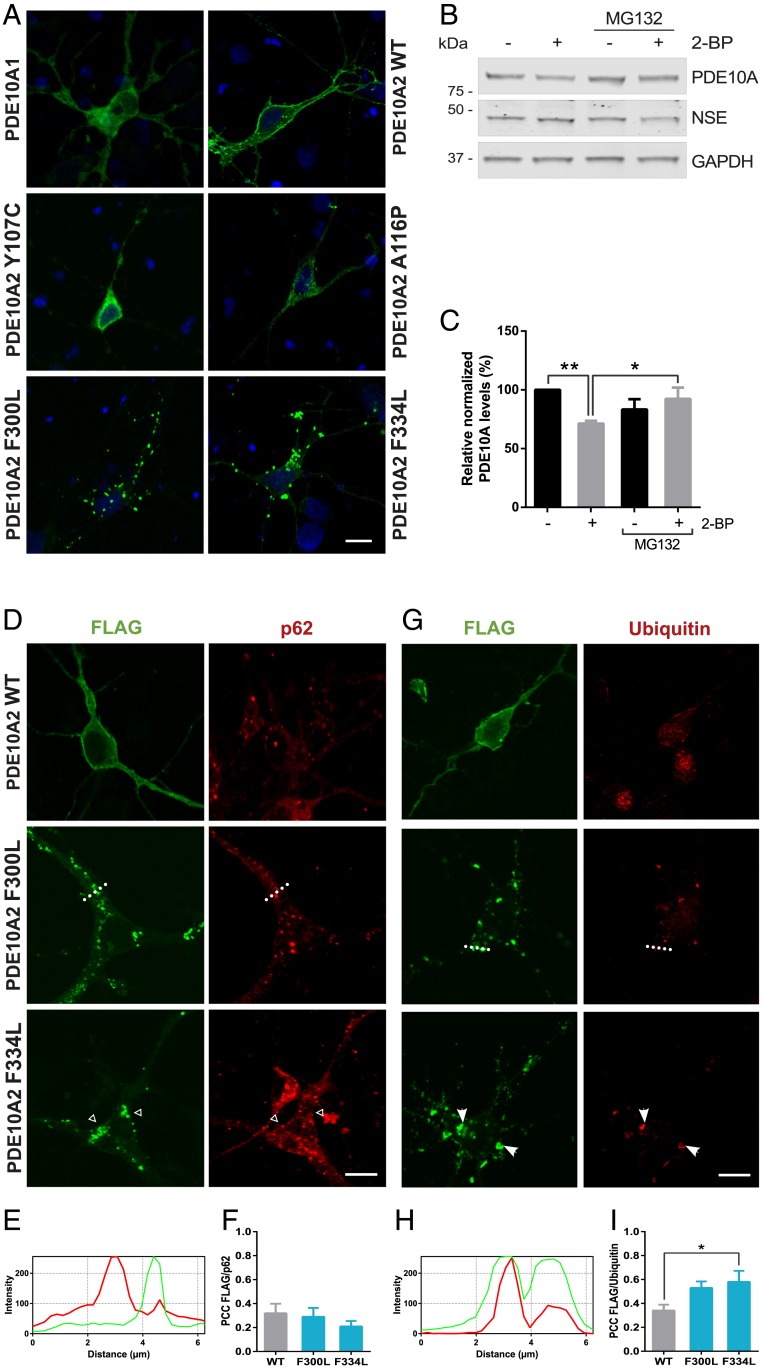
The aberrant localization of PDE10A GAF mutant proteins is replicated in striatal neurons. (*A*) Analysis of the different PDE10A-FLAG variants in transfected rat primary striatal neurons (FLAG in green and DAPI in blue). (Scale bar, 10 μm.) (*B*) Analysis of PDE10A turnover after palmitoylation inhibition in striatal neurons. Cells were treated with 100 µM 2-BP for 24 h and 20 µM MG132 for 5 h. (*C*) Quantification of PDE10A levels after palmitoylation and/or proteasome inhibition in striatal neurons. Data normalized by neuron-specific enolase (NSE) levels is expressed as mean ± SEM relative to an untreated condition and statistical analysis was performed by one-way ANOVA followed by Bonferroni’s post hoc test (**P = 0.0024, *P = 0.0245; *n* = 5). (*D* and *G*) Colocalization analysis between the indicated PDE10A2 variants (green) and p62 (*D*) or ubiquitin (*G*) in rat striatal neurons. Arrows indicate the presence of ubiquitin in PDE10A aggresomes and empty arrows show lack of recruitment of p62 to these neuronal aggregates. (*E* and *H*) Line intensity profiles corresponding to the section shown in neurons expressing the PDE10A2 F300L form (dotted line). (*F* and *I*) PCC quantification of the indicated PDE10A2-FLAG forms with p62 (*F*) or ubiquitin (*I*). Statistical significance was evaluated with one-way ANOVA followed by Dunnett’s post hoc test (*P = 0.0458; *n* = 9).

To further validate our data from model cell lines, we also investigated rat primary striatal neuronal cultures expressing the PDE10A constructs. Consistent with the distribution pattern in cell lines, PDE10A2 Y107C and A116P showed a cytosolic localization similar to PDE10A1 (Fig. 6*A*). Furthermore, treatment with palmitoylation inhibitor 2-bromopalmitate (2-BP), which promotes cytosolic distribution (19), reduced endogenous levels of PDE10A in striatal neurons, a decrease that can be prevented by proteasomal inhibition (Fig. 6 *B* and *C*). F300L and F334L mutants accumulated in aggregates in contrast to the strong membrane immunostaining observed for the WT form. Interestingly, the size of aggregates in striatal neurons was 10 times smaller than in HEK293 cells, with an average diameter of 1.3 µm, similar to the ones observed in the neuron-like cells PC12 and SH-SY5Y (*SI Appendix*, Fig. S4*A*). Moreover, neuronal cultures presented a more dispersed distribution of the aggregates throughout the cell. It has been previously described that the overall structure of the aggresomes varies depending on the cell system and the phase of formation when they are studied as they can fuse to each other (46). This cellular discrepancy was extended to the components that are recruited into the PDE10A aggregates. Thus, p62 immunostaining did not colocalize with the labeled aggregates in striatal neurons (Fig. 6 *D*–*F*), although ubiquitinated proteins were still accumulated in these inclusions (Fig. 6 *G*–*I*). Similarly, relocation of the PDE10A2 mutant aggresomes to EEA1 was slightly reduced compared to HEK293 cells (*SI Appendix*, Fig. S4*B*), while association of these structures with the P-body marker Dcp1a was increased in the neuronal cultures (*SI Appendix*, Fig. S4 *F* and *G*). Nonetheless, LAMP1 could also be detected in PDE10A2 F300L and F334L inclusions (*SI Appendix*, Fig. S4*C*), suggesting an autophagic route for these aggresomes in neurons. No colocalization with HDAC6 or Golgi and endoplasmic reticulum markers was observed (*SI Appendix*, Fig. S4 *D*–*F*).

## Discussion

PDE10A has emerged as a central regulator of motor control (47) following the identification of single mutations in the enzyme that lead to hyperkinetic movement disorders (48). Here, we show that the underlying mechanisms of these disease-associated mutations in PDE10A are related to abnormal subcellular localization and aberrant degradation of the enzyme, which ultimately disrupts the precise balance of compartmentalized cAMP signaling. The pathological phenotype observed in patients correlates to the impairment of the most abundant striatal splice variant, the membrane-bound PDE10A2 (21). Trafficking to the plasma membrane is achieved by palmitoylation on the isoform-specific N-terminal region, although PKA phosphorylation is able to prevent translocation (19) and maintain a small pool of cytosolic PDE10A2. We have shown that substitutions Y107C and A116P located in the GAF-A domain prevent trafficking to the membrane in cell lines and primary striatal neurons. While the GAF-B domain is responsible for cyclic nucleotide binding and dimerization (49), the function of the GAF-A region in PDE10A remains elusive. Additionally, GAF domains in other PDEs have also been implicated in protein–protein interactions (50). This work shows that GAF-A mutations abolish palmitoylation of PDE10A. Therefore, it could be possible that Y107C and A116P mutations are situated in the binding region for the palmitoyl transferase (zDHHC), as some zDHHC substrates contain the interacting site distant from the palmitoylated residues (51). It is also important to note that these PDE10A2 mutants retain the ability to hydrolyze cAMP in a similar manner to the cytosolic PDE10A1. Thus, the diminished PDE10A activity at the plasma membrane associated with the motor disorder, reinforces the paradigm of compartmentalized cAMP dynamics being crucial for normal cell function (52). This has been exemplified before with the displacement of PDE4D7 from the plasma membrane, leading to aberrant cAMP signaling and an increase in the proliferation of prostate cancer cells (53). The shift of PDE10A2 GAF-A mutants to the cytosol also increases the turnover rate compared to the membrane-bound form. Indeed, they exhibit similar vulnerability to proteasomal degradation as PDE10A1, which agrees with the predominant action of the UPS pathway on cytosolic proteins (54). In fact, the elevation of the proteasomal activity by overexpression of NUB1 has been shown to decrease PDE10A levels in an HD mouse model (22). In contrast, the PDE10A2 membrane-bound forms are degraded by the autophagy system, in a similar fashion to most synaptic proteins (29, 55) and a previously suggested route for PDE10A (23). The decline in cAMP degradation observed with the GAF-A mutants would enhance PKA activity, which could phosphorylate the proteasome component Rpt6, promote UPS function, and decrease PDE10A levels even more (56). This faster turnover would explain the reduced levels of PDE10A detected in patients carrying the recessive mutation Y107C (13). Furthermore, the complete lack of PDE10A at the synapse could be responsible for the earlier clinical manifestations observed in these patients compared to those carrying the GAF-B mutations. Interestingly, patients with biallelic mutations in the active site of the catalytic domain of PDE10A (L675P) also showed an early manifestation of hyperkinetic movements (57).

A decrease in PDE10A expression has also been observed in the striatum of individuals harboring the substitution F300L (17). We have shown here that PDE10A protein carrying the dominant mutations F300L and F334L generate aggregates in the cytosol that reduce its availability at the plasma membrane. Nonetheless, these substitutions do not affect the intrinsic PDE activity of PDE10A at the inner surface of the cell, although a lower level of the enzyme at the plasma membrane may disturb cAMP signaling and would probably alter cyclic nucleotide dynamics in medium spiny neurons. The pathogenic effect of the mutations in the GAF-B domain of PDE10A could further be increased with the presence of aggregates resulting from improperly folded proteins. Aggregation can sometimes occur when protein expression rates are too high, causing an overload on the folding machinery (58). This phenomenon is one of the limitations of the present study and possibly the reason why a small proportion of HEK293 cells showed aggregates of the PDE10A2 WT, which was not mirrored in striatal neurons. Conversely, the high incidence of protein misfolding with the F300L and F334L forms indicate that their abnormal association is induced by these mutations. Both mutations affect highly conserved aromatic residues located in the cAMP binding pocket. The conformational view of this GAF-B region suggests that these 2 phenylalanines are implicated in an edge-to-face interaction and their substitution for a nonaromatic amino acid could destabilize the domain and the folding of the protein. Actually, aromatic pairs usually result in stabilizing such scaffolds (59) and a computational analysis found that aggregation-prone sequences normally contained a pair of aromatic residues, and at least one phenylalanine (60). These PDE10A2 F300L and F334L aggregates cluster into specialized organelles called aggresomes, which are large structures that sequester aggregated proteins to minimize obstruction of cellular processes and deliver them to lysosomes for degradation (37). Aggresome formation is a dynamic process that can vary depending on the cell type (46, 61, 62), explaining the differences in size and components of the PDE10A inclusions between the different cellular systems.

Particularly interesting is the sequestration of the autophagy-adapter protein p62 into the PDE10A aggresomes in HEK293 cells as opposed to its absence in the neuronal aggregates. Considering the smaller volume and dispersion of the PDE10A in striatal inclusions, the lack of association with p62 could correspond to an earlier accumulation stage or the implication of a different receptor for lysosomal degradation, such as NBR1 (63). The function of the aggresomal structures is now assumed to be a protective response against the toxic misfolded proteins and the formation of these inclusions is dissociated from cell death (64–66). Accordingly, the presence of the PDE10A F300L or F334L aggregates did not reduce cellular viability and, indeed, these cells experienced a slower stress-induced apoptosis, possibly due to an already impaired ALP that attenuates arsenite-induced autophagic death. Aggresomes are a hallmark of many neurodegenerative diseases where it is believed that the progressive accumulation of misfolded proteins leads to cellular dysfunction, loss of synaptic connections, and selective brain damage (67). Although data from overexpression models should be approached with caution and patient-derived systems are needed to corroborate misfolding and aggregation of these mutants, it is plausible that a common pathological mechanism occurs after expression of the aggregated-prone PDE10A forms. Indeed, the PDE10A dominant mutations F300L and F334L, unlike the mutations in the GAF-A domain, severely affect striatal structure as shown by MRI scans (15).

PDE10A has been proposed as a novel therapeutic target for diseases of the basal ganglia but inhibitors of this enzyme failed to show efficacy in clinical trials for both HD and schizophrenia (https://www.clinicaltrials.gov/: NCT02197130, NCT02074410, NCT02342548, NCT01806896, NCT01939548, NCT01175135). Our results show that an aberrant compartmentalization of PDE10A underlies the hyperkinetic movement disorders observed in individuals carrying mutations in this gene. Consequently, the development of small molecules directed against specific and highly localized PDE10A pools, such as agents targeting the binding between the enzyme and zDHHCs or members of the ALP or UPS, could be of interest from a therapeutic perspective.

## Materials and Methods

### Culture and Transfection of HEK293 Cells and Primary Striatal Neurons.

HEK293 cells were sourced from ATCC (CRL-1573) and primary striatal cultures were collected from embryonic day 17 Sprague–Dawley rats (68). Cells were transfected using Lipofectamine LTX with Plus reagent (ThermoFisher Scientific) according to the manufacturer’s instructions. For further details, see *SI Appendix*, *Supplementary Methods*.

### Animals.

All animal procedures were performed in compliance with European Union Directive 2010/63/EU and Animal (Scientific Procedures) Act 1986 with ethical review approval (University of Glasgow). Sprague–Dawley rats were obtained from Charles River Laboratories and bred in our facilities. Animals were housed in groups of 1 to 2 pregnant rats on a 12-h light/dark cycle with food and water available ad libitum. All efforts were made to minimize animal suffering and reduce the number of animals killed.

### Immunoblot Analysis.

Preparation of protein extracts, fractionation and immunoblot analysis is detailed in *SI Appendix*, *Supplementary Methods*.

### Immunocytochemistry.

Details regarding immunofluorescence can be found in *SI Appendix*, *Supplementary Methods*.

### Structural GAF-B Simulation.

The mutations F300L and F334L, respectively, were introduced to the known GAF-B structure of PDE10A (PDB ID code 2ZMF). Both mutated structures were then minimized using the molecular operating environment (MOE) without any restrictions (https://www.chemcomp.com/Products.htm).

### PDE Assay.

PDE activity was measured using a radioactive cAMP hydrolysis assay as described previously (69). Data dose–response curves and IC_50_ values were calculated using GraphPad Prism. For further details, see *SI Appendix*, *Supplementary Methods*.

### Electrophysiology.

Details on whole cell patch-clamp technique for analysis of PDE10A modulation of CNG-mediated currents can be found in *SI Appendix*, *Supplementary Methods*.

### FRET Imaging.

FRET imaging experiments were performed 24 to 48 h after transfection of HEK293 cells as previously described (53), with the cytosolic EPAC-1 cAMP sensor (28) or a modified version encoding the FYXD1 subunit of Na^2+^/K^+^ ATPase in the N-terminal region to direct the probe to the plasma membrane. For additional details, see *SI Appendix*, *Supplementary Methods*.

### Real-Time Cell Monitoring.

Cell survival was monitored in real-time using the electrical impedance‐based xCELLigence system (ACEA Biosciences), as detailed in *SI Appendix*, *Supplementary Methods*.

### Statistical Analysis.

All data were expressed as mean ± SEM of at least 3 independent experiments. Statistical significance was determined by a one-way ANOVA with Dunnett’s post hoc correction or two-way ANOVA followed by a Tukey’s or Bonferroni’s post hoc test. A *P* value smaller than 0.05 was considered statistically significant (*P < 0.05, **P < 0.01, ***P < 0.001). Statistical analysis was performed using GraphPad Prism software.

### Data and Materials Availability.

All data used in the paper are present in the main text and *SI Appendix*.

## Supplementary Material

Supplementary File

## References

[1] Nicola S. M., The nucleus accumbens as part of a basal ganglia action selection circuit. Psychopharmacology (Berl.) 191, 521–550 (2007). 16983543 10.1007/s00213-006-0510-4

[2] Han I., You Y., Kordower J. H., Brady S. T., Morfini G. A., Differential vulnerability of neurons in Huntington’s disease: The role of cell type-specific features. J. Neurochem. 113, 1073–1091 (2010). 20236390 10.1111/j.1471-4159.2010.06672.x PMC2890032

[3] Hu H., McCaw E. A., Hebb A. L., Gomez G. T., Denovan-Wright E. M., Mutant huntingtin affects the rate of transcription of striatum-specific isoforms of phosphodiesterase 10A. Eur. J. Neurosci. 20, 3351–3363 (2004). 15610167 10.1111/j.1460-9568.2004.03796.x

[4] Ahmad R., PET imaging shows loss of striatal PDE10A in patients with Huntington disease. Neurology 82, 279–281 (2014). 24353339 10.1212/WNL.0000000000000037

[5] Wilson H., Loss of extra-striatal phosphodiesterase 10A expression in early premanifest Huntington’s disease gene carriers. J. Neurol. Sci. 368, 243–248 (2016). 27538642 10.1016/j.jns.2016.07.033

[6] Niccolini F., Loss of phosphodiesterase 10A expression is associated with progression and severity in Parkinson’s disease. Brain 138, 3003–3015 (2015). 26210536 10.1093/brain/awv219

[7] Niccolini F., Altered PDE10A expression detectable early before symptomatic onset in Huntington’s disease. Brain 138, 3016–3029 (2015). 26198591 10.1093/brain/awv214

[8] Russell D. S., Change in PDE10 across early Huntington disease assessed by [18F]MNI-659 and PET imaging. Neurology 86, 748–754 (2016). 26802091 10.1212/WNL.0000000000002391

[9] Hebb A. L., Robertson H. A., Denovan-Wright E. M., Striatal phosphodiesterase mRNA and protein levels are reduced in Huntington’s disease transgenic mice prior to the onset of motor symptoms. Neuroscience 123, 967–981 (2004). 14751289 10.1016/j.neuroscience.2003.11.009

[10] Ooms M., Early decrease of type 1 cannabinoid receptor binding and phosphodiesterase 10A activity in vivo in R6/2 Huntington mice. Neurobiol. Aging 35, 2858–2869 (2014). 25018107 10.1016/j.neurobiolaging.2014.06.010

[11] Wood H., Neurodegenerative disease: Changes in brain phosphodiesterase 10A levels in neurodegenerative basal ganglia disorders. Nat. Rev. Neurol. 11, 483 (2015). 26260657 10.1038/nrneurol.2015.148

[12] Russell D. S., The phosphodiesterase 10 positron emission tomography tracer, [18F]MNI-659, as a novel biomarker for early Huntington disease. JAMA Neurol. 71, 1520–1528 (2014). 25322077 10.1001/jamaneurol.2014.1954

[13] Diggle C. P., Biallelic mutations in PDE10A lead to loss of striatal PDE10A and a hyperkinetic movement disorder with onset in infancy. Am. J. Hum. Genet. 98, 735–743 (2016). 27058446 10.1016/j.ajhg.2016.03.015 PMC4833436

[14] Esposito S., A PDE10A de novo mutation causes childhood-onset chorea with diurnal fluctuations. Mov. Disord. 32, 1646–1647 (2017). 28949041 10.1002/mds.27175

[15] Mencacci N. E., De novo mutations in PDE10A cause childhood-onset chorea with bilateral striatal lesions. Am. J. Hum. Genet. 98, 763–771 (2016). 27058447 10.1016/j.ajhg.2016.02.015 PMC4833291

[16] Narayanan D. L., Deshpande D., Das Bhowmik A., Varma D. R., Dalal A., Familial choreoathetosis due to novel heterozygous mutation in PDE10A. Am. J. Med. Genet. A. 176, 146–150 (2018). 29130591 10.1002/ajmg.a.38507

[17] Niccolini F., PDE10A and ADCY5 mutations linked to molecular and microstructural basal ganglia pathology. Mov. Disord. 33, 1961–1965 (2018). 30345538 10.1002/mds.27523

[18] Russwurm C., Koesling D., Russwurm M., Phosphodiesterase 10A is tethered to a synaptic signaling complex in striatum. J. Biol. Chem. 290, 11936–11947 (2015). 25762721 10.1074/jbc.M114.595769 PMC4424333

[19] Charych E. I., Jiang L. X., Lo F., Sullivan K., Brandon N. J., Interplay of palmitoylation and phosphorylation in the trafficking and localization of phosphodiesterase 10A: Implications for the treatment of schizophrenia. J. Neurosci. 30, 9027–9037 (2010). 20610737 10.1523/JNEUROSCI.1635-10.2010 PMC6632485

[20] Loughney K., Isolation and characterization of PDE10A, a novel human 3′, 5′-cyclic nucleotide phosphodiesterase. Gene 234, 109–117 (1999). 10393245 10.1016/s0378-1119(99)00171-7

[21] Kotera J., Subcellular localization of cyclic nucleotide phosphodiesterase type 10A variants, and alteration of the localization by cAMP-dependent protein kinase-dependent phosphorylation. J. Biol. Chem. 279, 4366–4375 (2004). 14604994 10.1074/jbc.M308471200

[22] Vodicka P., Effects of exogenous NUB1 expression in the striatum of HDQ175/Q7 mice. J. Huntingtons Dis. 5, 163–174 (2016). 27314618 10.3233/JHD-160195

[23] Vodicka P., Autophagy activation by transcription factor EB (TFEB) in striatum of HDQ175/Q7 mice. J. Huntingtons Dis. 5, 249–260 (2016). 27689619 10.3233/JHD-160211 PMC5088406

[24] Price J. C., Guan S., Burlingame A., Prusiner S. B., Ghaemmaghami S., Analysis of proteome dynamics in the mouse brain. Proc. Natl. Acad. Sci. U.S.A. 107, 14508–14513 (2010). 20699386 10.1073/pnas.1006551107 PMC2922600

[25] Rich T. C., Tse T. E., Rohan J. G., Schaack J., Karpen J. W., In vivo assessment of local phosphodiesterase activity using tailored cyclic nucleotide-gated channels as cAMP sensors. J. Gen. Physiol. 118, 63–78 (2001). 11429444 10.1085/jgp.118.1.63 PMC2233745

[26] Rich T. C., A uniform extracellular stimulus triggers distinct cAMP signals in different compartments of a simple cell. Proc. Natl. Acad. Sci. U.S.A. 98, 13049–13054 (2001). 11606735 10.1073/pnas.221381398 PMC60822

[27] Rich T. C., Cellular mechanisms underlying prostaglandin-induced transient cAMP signals near the plasma membrane of HEK-293 cells. Am. J. Physiol. Cell Physiol. 292, C319–C331 (2007). 16899551 10.1152/ajpcell.00121.2006 PMC4712347

[28] Nikolaev V. O., Bünemann M., Hein L., Hannawacker A., Lohse M. J., Novel single chain cAMP sensors for receptor-induced signal propagation. J. Biol. Chem. 279, 37215–37218 (2004). 15231839 10.1074/jbc.C400302200

[29] Jin E. J., Kiral F. R., Hiesinger P. R., The where, what, and when of membrane protein degradation in neurons. Dev. Neurobiol. 78, 283–297 (2018). 28884504 10.1002/dneu.22534 PMC5816708

[30] Li Y., Martin B. R., Cravatt B. F., Hofmann S. L., DHHC5 protein palmitoylates flotillin-2 and is rapidly degraded on induction of neuronal differentiation in cultured cells. J. Biol. Chem. 287, 523–530 (2012). 22081607 10.1074/jbc.M111.306183 PMC3249106

[31] Hyttinen J. M., Clearance of misfolded and aggregated proteins by aggrephagy and implications for aggregation diseases. Ageing Res. Rev. 18, 16–28 (2014). 25062811 10.1016/j.arr.2014.07.002

[32] Grumati P., Dikic I., Ubiquitin signaling and autophagy. J. Biol. Chem. 293, 5404–5413 (2018). 29187595 10.1074/jbc.TM117.000117 PMC5900779

[33] Bjørkøy G., p62/SQSTM1 forms protein aggregates degraded by autophagy and has a protective effect on huntingtin-induced cell death. J. Cell Biol. 171, 603–614 (2005). 16286508 10.1083/jcb.200507002 PMC2171557

[34] Gomez L. L., Alam S., Smith K. E., Horne E., Dell’Acqua M. L., Regulation of A-kinase anchoring protein 79/150-cAMP-dependent protein kinase postsynaptic targeting by NMDA receptor activation of calcineurin and remodeling of dendritic actin. J. Neurosci. 22, 7027–7044 (2002). 12177200 10.1523/JNEUROSCI.22-16-07027.2002 PMC6757891

[35] Razi M., Chan E. Y., Tooze S. A., Early endosomes and endosomal coatomer are required for autophagy. J. Cell Biol. 185, 305–321 (2009). 19364919 10.1083/jcb.200810098 PMC2700373

[36] Olzmann J. A., Li L., Chin L. S., Aggresome formation and neurodegenerative diseases: Therapeutic implications. Curr. Med. Chem. 15, 47–60 (2008). 18220762 10.2174/092986708783330692 PMC4403008

[37] Johnston J. A., Ward C. L., Kopito R. R., Aggresomes: A cellular response to misfolded proteins. J. Cell Biol. 143, 1883–1898 (1998). 9864362 10.1083/jcb.143.7.1883 PMC2175217

[38] Kawaguchi Y., The deacetylase HDAC6 regulates aggresome formation and cell viability in response to misfolded protein stress. Cell 115, 727–738 (2003). 14675537 10.1016/s0092-8674(03)00939-5

[39] Waelter S., Accumulation of mutant huntingtin fragments in aggresome-like inclusion bodies as a result of insufficient protein degradation. Mol. Biol. Cell 12, 1393–1407 (2001). 11359930 10.1091/mbc.12.5.1393 PMC34592

[40] Wolozin B., Regulated protein aggregation: Stress granules and neurodegeneration. Mol. Neurodegener. 7, 56 (2012). 23164372 10.1186/1750-1326-7-56 PMC3519755

[41] Lee K., β-Catenin nuclear translocation in colorectal cancer cells is suppressed by PDE10A inhibition, cGMP elevation, and activation of PKG. Oncotarget 7, 5353–5365 (2016). 26713600 10.18632/oncotarget.6705 PMC4868691

[42] Selkoe D. J., Folding proteins in fatal ways. Nature 426, 900–904 (2003). 14685251 10.1038/nature02264

[43] Swart C., Neurodegenerative disorders: Dysregulation of a carefully maintained balance? Exp. Gerontol. 58, 279–291 (2014). 25219768 10.1016/j.exger.2014.09.003

[44] Bai J., Taurine protects against As2O3-induced autophagy in livers of rat offsprings through PPARγ pathway. Sci. Rep. 6, 27733 (2016). 27291853 10.1038/srep27733 PMC4904213

[45] Qiu T., Taurine attenuates arsenic-induced pyroptosis and nonalcoholic steatohepatitis by inhibiting the autophagic-inflammasomal pathway. Cell Death Dis. 9, 946 (2018). 30237538 10.1038/s41419-018-1004-0 PMC6148242

[46] Garcia-Mata R., Gao Y. S., Sztul E., Hassles with taking out the garbage: Aggravating aggresomes. Traffic 3, 388–396 (2002). 12010457 10.1034/j.1600-0854.2002.30602.x

[47] Schülke J. P., Brandon N. J., Current understanding of PDE10A in the modulation of basal ganglia circuitry. Adv. Neurobiol. 17, 15–43 (2017). 28956328 10.1007/978-3-319-58811-7_2

[48] Whiteley E. L., Tejeda G. S., Baillie G. S., Brandon N. J., PDE10A mutations help to unwrap the neurobiology of hyperkinetic disorders. Cell. Signal. 60, 31–38 (2019). 30951862 10.1016/j.cellsig.2019.04.001

[49] Handa N., Crystal structure of the GAF-B domain from human phosphodiesterase 10A complexed with its ligand, cAMP. J. Biol. Chem. 283, 19657–19664 (2008). 18477562 10.1074/jbc.M800595200

[50] Matte S. L., Laue T. M., Cote R. H., Characterization of conformational changes and protein-protein interactions of rod photoreceptor phosphodiesterase (PDE6). J. Biol. Chem. 287, 20111–20121 (2012). 22514270 10.1074/jbc.M112.354647 PMC3370194

[51] Lemonidis K., Sanchez-Perez M. C., Chamberlain L. H., Identification of a novel sequence motif recognized by the ankyrin repeat domain of zDHHC17/13 S-acyltransferases. J. Biol. Chem. 290, 21939–21950 (2015). 26198635 10.1074/jbc.M115.657668 PMC4571948

[52] Baillie G. S., Compartmentalized signalling: Spatial regulation of cAMP by the action of compartmentalized phosphodiesterases. FEBS J. 276, 1790–1799 (2009). 19243430 10.1111/j.1742-4658.2009.06926.x

[53] Henderson D. J., The cAMP phosphodiesterase-4D7 (PDE4D7) is downregulated in androgen-independent prostate cancer cells and mediates proliferation by compartmentalising cAMP at the plasma membrane of VCaP prostate cancer cells. Br. J. Cancer 110, 1278–1287 (2014). 24518597 10.1038/bjc.2014.22 PMC3950871

[54] Bhattacharyya S., Yu H., Mim C., Matouschek A., Regulated protein turnover: Snapshots of the proteasome in action. Nat. Rev. Mol. Cell Biol. 15, 122–133 (2014). 24452470 10.1038/nrm3741 PMC4384331

[55] Hakim V., Cohen L. D., Zuchman R., Ziv T., Ziv N. E., The effects of proteasomal inhibition on synaptic proteostasis. EMBO J. 35, 2238–2262 (2016). 27613546 10.15252/embj.201593594 PMC5069550

[56] Lin J. T., Regulation of feedback between protein kinase A and the proteasome system worsens Huntington’s disease. Mol. Cell. Biol. 33, 1073–1084 (2013). 23275441 10.1128/MCB.01434-12 PMC3623082

[57] Knopp C., PDE10A mutation in two sisters with a hyperkinetic movement disorder—Response to levodopa. Parkinsonism Relat. Disord. 63, 240–242 (2019). 30777652 10.1016/j.parkreldis.2019.02.007

[58] Halff E. F., Versteeg M., Brondijk T. H., Huizinga E. G., When less becomes more: Optimization of protein expression in HEK293-EBNA1 cells using plasmid titration—A case study for NLRs. Protein Expr. Purif. 99, 27–34 (2014). 24680733 10.1016/j.pep.2014.03.010

[59] Madhusudan Makwana K., Mahalakshmi R., Implications of aromatic-aromatic interactions: From protein structures to peptide models. Protein Sci. 24 1920–1933 (2015). 26402741 10.1002/pro.2814 PMC4815235

[60] Frederix P. W., Exploring the sequence space for (tri-)peptide self-assembly to design and discover new hydrogels. Nat. Chem. 7, 30–37 (2015). 25515887 10.1038/nchem.2122

[61] García-Mata R., Bebök Z., Sorscher E. J., Sztul E. S., Characterization and dynamics of aggresome formation by a cytosolic GFP-chimera. J. Cell Biol. 146, 1239–1254 (1999). 10491388 10.1083/jcb.146.6.1239 PMC2156127

[62] Tiwari A., Caveolin-1 is an aggresome-inducing protein. Sci. Rep. 6, 38681 (2016). 27929047 10.1038/srep38681 PMC5144149

[63] Kirkin V., A role for NBR1 in autophagosomal degradation of ubiquitinated substrates. Mol. Cell 33, 505–516 (2009). 19250911 10.1016/j.molcel.2009.01.020

[64] Arrasate M., Mitra S., Schweitzer E. S., Segal M. R., Finkbeiner S., Inclusion body formation reduces levels of mutant huntingtin and the risk of neuronal death. Nature 431, 805–810 (2004). 15483602 10.1038/nature02998

[65] Saudou F., Finkbeiner S., Devys D., Greenberg M. E., Huntingtin acts in the nucleus to induce apoptosis but death does not correlate with the formation of intranuclear inclusions. Cell 95, 55–66 (1998). 9778247 10.1016/s0092-8674(00)81782-1

[66] Tanaka M., Aggresomes formed by alpha-synuclein and synphilin-1 are cytoprotective. J. Biol. Chem. 279, 4625–4631 (2004). 14627698 10.1074/jbc.M310994200

[67] Ross C. A., Poirier M. A., Protein aggregation and neurodegenerative disease. Nat. Med. 10 (suppl.), S10–S17 (2004). 15272267 10.1038/nm1066

[68] Falk T., Zhang S., Erbe E. L., Sherman S. J., Neurochemical and electrophysiological characteristics of rat striatal neurons in primary culture. J. Comp. Neurol. 494, 275–289 (2006). 16320238 10.1002/cne.20819 PMC2923039

[69] Marchmont R. J., Houslay M. D., A peripheral and an intrinsic enzyme constitute the cyclic AMP phosphodiesterase activity of rat liver plasma membranes. Biochem. J. 187, 381–392 (1980). 6249268 10.1042/bj1870381 PMC1161804

