# Supplementary material for: Chorea-related mutations in PDE10A result in aberrant compartmentalization and functionality of the enzyme
Source: Proc Natl Acad Sci U S A. 2019 Dec 23;117(1):677–88. doi: 10.1073/pnas.1916398117 (PMC6955301; doi:10.1073/pnas.1916398117)

Chorea-related mutations in PDE10A result in aberrant compartmentalization and functionality of the enzyme

Gonzalo S. Tejeda, Ellanor L. Whiteley, Tarek Z. Deeb, Roland W. Bürli, Stephen J. Moss, Eamonn Sheridan, Nicholas J. Brandon and George S. Baillie

Table of contents

Appendix Supplementary Methods

Appendix Table S1: Plasmids

Appendix Table S2: Primary and secondary antibodies

Appendix Figure S1: Distribution and expression of PDE10A variants in different cell lines.

Appendix Figure S2: Analysis of the subcellular localization of PDE10A2 GAF-B mutants.

Appendix Figure S3: Characterization of PDE10A2 GAF-B aggregates in HEK293 cells.

Appendix Figure S4: Characterization of PDE10A2 GAF-B aggregates in rat striatal neurons.

Appendix Supplementary Methods

Culture of primary striatal neurons

Brains were immediately placed in ice cold HBSS (ThermoFisher Scientific) and the striata were isolated under a dissecting microscope. After dissection, striatal cells were mechanically dissociated by gentle trituration in dissection buffer and cells were pelleted by centrifugation at 1000 x g for 5 minutes and resuspended in 5 ml of plating media (high-glucose DMEM supplemented with 10% FBS and 1% P/S). The cell suspension was seeded at a density of 0.5 x 10^6 cells/ml on 24 well plates with 13 mm glass coverslips previously treated with poly-L-lysine (100 µg/ml, P0899, Sigma-Aldrich) overnight at 37 °C. The following day the media was removed and replaced with Neurobasal containing B27 supplement (ThermoFisher Scientific), 2 mM glutamax and 1% P/S. Half of the media was changed every 4 days and 5 µM of cytosine β-D-arabinofuranoside (Ara-C, Sigma-Aldrich) was added on the fourth day to control the proliferation of non-neuronal cells. Neurons were treated with 2-bromopalmitate (Sigma-Aldrich) or transfected with the indicated cDNA constructs at 12 days in vitro (DIVs).

Culture of cell lines

HEK293 cells were cultured in high-glucose Dulbecco's Modified Eagle's Medium (DMEM) containing 10% foetal bovine serum (FBS), 2 mm L-glutamine, 1% non-essential amino acids, 100 U/ml penicillin and 100 µg/ml streptomycin (1% P/S) and incubated at 37 °C in a humidified atmosphere with 5% CO2. PC12 cells were grown in high-glucose

www.pnas.org/cgi/doi/10.1073/pnas.1916398116

DMEM medium containing 10% FBS, 5% horse serum, 2 mm L-glutamine 1% P/S while SH-SY5Y cells were maintained in high-glucose DMEM and F12-Ham's media at a 1:1 ratio, supplemented with 10% FBS, 2 mm L-glutamine 1% P/S. Cells were incubated at 37 °C in a humidified atmosphere with 5% CO2 and transfected with the indicated cDNA constructs using Lipofectamine LTX with Plus reagent according to the manufacturer's instructions. HEK293 cells were treated 24 h after transfection with the pharmacological agents MG132 (Millipore), CQ (Sigma-Aldrich), forskolin or rolipram (Enzo Life Sciences) as described in the text.

Preparation of protein extracts and immunoblot analysis

HEK293 cells were lysed in 3T3 buffer (50 mM NaCl, 50 mM NaF, 25 mM HEPES, 5 mM EDTA, 30 mM sodium pyrophosphate, 10% glycerol, 1% Triton X-100; pH 7.5) and striatal neurons in RIPA buffer (10 mM Tris-Cl, 1 mM EDTA, 1% Triton X-100, 0.1% sodium deoxycholate, 0.1% SDS, 140 mM NaCl, pH 7.5) supplemented with protease inhibitor tablets (Roche) and incubated for 30 min at 4 °C. Lysates were first centrifuged at 1,000 x g for 5 min. Supernatants were recovered and detergent insoluble proteins were precipitated by centrifugation at 10,000 × g for 10 min, retaining the soluble fraction. Pellets were resuspended by brief sonication in an identical volume of 3T3 lysis buffer with 1% SDS, yielding the insoluble fraction. Equal concentrations of protein samples were boiled in SDS loading buffer (10% SDS, 300mM Tris-HCl pH 7.2, 0.05% bromothymol blue, 10% β-mercaptoethanol) and 20–40 µg of each cell lysate were resolved on NuPAGE precast 4-12% gels (Invitrogen) followed by transfer to nitrocellulose membranes (Whatman). Ponceau-S staining (0.2% Ponceau-S red, 1% acetic acid) was performed to verify proper transfer of the proteins and the membranes were then blocked in 5% non-fat dry milk (Marvel) in TBST (25 mM Tris-HCl; pH 7.6, 100 mM NaCl, 0.5% Tween 20) for 1 h at room temperature. Membranes were incubated with the different primary antibodies in blocking solution overnight at 4 °C. Antibodies used in this study are detailed in Appendix Table 2. After washing three times for 5 min each with TBST, appropriate secondary antibodies were added to the membranes for 1 h at RT. Immunoreactive bands were visualized using the Licor Odyssey system and densitometry of fluorescence was measured using Image Studio Lite (Licor Biosciences).

Immunoprecipitation

Approximately 1 mg of cell lysates pre-cleared with Protein G sepharose beads (ThermoFisher Scientific) for 30 min at 4 °C and then co-immunoprecipitated overnight at 4 °C with 1 µg anti-Ubiquitin (sc-8017, Santa Cruz Biotechnology) combined with 25 µl of 50% protein G sepharose beads. Samples were washed six times with 3T3 lysis buffer and eluted with SDS loading buffer. Equivalent volumes of the immunoprecipitated complexes were analysed by immunoblot as indicated.

Subcellular Fractionation

Cells were homogenised in fractionation buffer (20 mM HEPES, 10 mM KCl, 10

mM MgCl2, 1 mM EDTA, 1mM EGTA supplemented with 1 mM DTT and protease inhibitor cocktail tablets) and incubated on ice for 30 minutes. Homogenates were centrifuged at 10,000 x g for 5 min at 4°C and the pellet was discarded. The suspension was centrifuged again at 40,000 x g for 1 h, yielding a cytosolic fraction in the supernatant. Pellets were resuspended in fractionation buffer by passing through a 25-gauge needle 10 times and centrifuged again at 40,000 x g for 45 min. Membrane fraction contained in the pellet was resuspended by brief sonication in TBS with 0.1% SDS. Identical amounts of total protein from homogenate, membrane and cytosolic fractions were subjected to SDS-PAGE and Western blot analysis using the indicated antibodies.

Purification of Palmitoylated Proteins

Palmitoylated proteins from lysates of transfected HEK293 cells were purified using preequilibrated thiopropyl-Sepharose (GE Life Sciences) in the presence of neutral hydroxylamine after blockade of free thiols with methyl methanethiosulfonate, as described previously (1). Samples before purification, not captured or captured by thiopropyl-Sepharose beads (unfractionated, unbound and acylated respectively) were heated for 10 min at 60 °C in SDS-PAGE loading buffer supplemented with 100 mm DTT and equal volumes were analysed by immunoblot using the indicated antibodies.

PDE assay

Briefly, a 2 µM mixture of [^{3}H] cAMP (Perkin Elmer) and unlabelled cAMP in assay buffer (20 mm Tris, 10 mM $MgCl_2$, pH 7.4) was mixed with membrane fractions of transfected HEK293 cells in KHEM buffer (50 mM KCl, 50 mM HEPES pH 7.2, 10 mM EGTA, 1.9 mM $MgCl_2$) supplemented with protease and phosphatase inhibitor tablets. PDE10A activity was evaluated with 0.3 µM-3 mM of the selective inhibitor MP10. Reactions were then incubated at 30°C for 10 min and terminated by boiling the samples for 2 min at 95°C. After cooling the samples on ice, 25 *µ*l of venom from Crotalus attrox (Sigma) was added and the samples incubated at 30°C for a further 10 min. A measure of 400 *µ*l of a resin slurry (Dowex 1x8, pH 3, Sigma, 44340 as a 1:1:1 mixture of resin:water:ethanol) was added to each tube and vortexed several times over a 15 min period before being centrifuged at 13,000 × *g* at 4°C for 3 min. Then, 150 *µ*l of the supernatant fraction from each tube was placed in a corresponding scintillation vial containing 1 ml of scintillant and taken for counting on a beta counter.

Immunocytochemistry

HEK293 cells or primary rat striatal neurons were fixed 24 h after transfection with 4% paraformaldehyde for 1 h at room temperature (RT). Coverslips were then washed 3 times with PBS, followed by incubation for 1 h with blocking buffer (PBS supplemented with 1% BSA and 0.2% Triton X-100). Afterwards, cells were incubated with the indicated primary antibodies diluted in blocking solution overnight at 4°C in a humidity chamber. Detection was achieved using secondary antibodies conjugated to Alexa Fluor 488 or Alexa Fluor 546 (1:500, Life Technologies) and coverslips were mounted on glass slides using ProLong

Gold with DAPI (ThermoFisher Scientific). All confocal images are single sections acquired with an inverted Zeiss Pascal laser-scanning confocal microscope (LSM) 510 with a water immersion objective using the LSM image acquisition software. For further details regarding image analysis, see SI Appendix, Supplementary Methods.

Image analysis

Confocal images were analysed using ImageJ Fiji (NIH Image). The number of cells with aggregates was estimated counting about 100 transfected HEK293 cells for each indicated construct in 4 independent experiments. For measurements of PDE10A enrichment at the plasma membrane in transfected HEK293 cells, intensity values across the outer border of single cells were acquired by plot profile and the fluorescence within 1.2 μm of the plasma membrane was calculated as the percentage relative to the signal obtained in the whole cell. This thickness was selected as 55% of the intensity values of the plasma membrane marker Wheat Germ Agglutinin (WGA) was contained in this region. Plasma membrane fluorescence intensity was corrected by subtracting the values obtained from regions absent of signal and normalized by measuring the PDE10A signal in the whole cell. Colocalization analysis was performed by calculating Pearson's Correlation coefficients (PCC) using the Coloc 2 plugin for Fiji in single cells outlined with the polygon drawing tool. For each colocalization class, 5-40 cells from images of at least three independent experiments were used for quantification. Image intensity profiles were generated using the "Profile Plot" command of ImageJ. The size of the aggresomes was calculated using the "Analyze particles" tool considering aggregates with an area above 0.5 μm2 and a circularity between 0.2-1.2.

Electrophysiology

The whole cell patch-clamp technique was used to record CNG-mediated currents from HEK-293 cells expressing CNGA2 channels containing two mutations, C460W and E583M, that increase cAMP sensitivity and eliminate direct activation by NO, respectively (2, 3). Importantly, the whole-cell patch-clamp technique dialyze the cell, and so the CNG channels only respond to cAMP fluctuations in their immediate vicinity at the plasma membrane. These cells were also transfected with plasmids encoding AC3, GFP and the different PDE10A forms as indicated. The recording chamber was continuously perfused with bath saline (140 mM NaCl, 4 mM KCl, 10 mM glucose, 10 mM HEPES, 0.1 mM $MgCl_2$; pH 7.4) and the patch electrodes were filled with electrode solution (140 mM KCl, 0.5 mM $MgCl_2$, 10 mM HEPES, 5 mM Na_2ATP, 0.5 mM Na_2GTP, 1.1 mM EGTA). Cells were visualised using an inverted Nikon TS100 microscope equipped with an epifluorescence LED (Lumencor, SOLA Light Engine) and they were voltage-clamped at an electrode potential of -40 mV. An initial treatment with 10 μM rolipram in bath saline was applied until a stable baseline was achieved and then CNGA2 channels were activated by locally applying 5 μM forskolin. PDE10A activity at the plasma membrane was assessed using 300 nM MP10. Results were expressed as a percentage of the current relative to the amplitude obtained with a 10 mM $MgCl_2$ treatment, used as an indication of specific CNGA2-mediated currents. The cur-

rents were recorded at room temperature (RT) using an Axopatch 200B amplifier, low-pass filtered at 1 KHz, digitized at 10 KHz using a Digidata 1550 interface, and acquired using pCLAMP10 software (all from Molecular Devices).

FRET imaging

Cells were maintained at RT in a modified Ringer solution (125 mM NaCl, 20 mM Hepes, 1 mM Na_3PO_4, 5 mM KCl, 1 mM $MgSO_4$, 5.5 mM glucose, $CaCl_2$ 1 mM, pH 7.4) and imaged on an inverted microscope (Olympus IX71) using a 100x oil immersion objective (Zeiss). Cells were first treated with 5 µM forskolin to assess the enzyme's capacity to handle a submaximal cAMP concentration, followed by 300 nM MP10 to assess PDE10A activity. Changes in cAMP concentration were monitored using MetaFluor software (Molecular Devices) by measuring background-subtracted 480/535 nm fluorescence emission values upon excitation of the transfected cells at 440 nm. FRET change was calculated as R/R0, where R is the ratio at time t and R0 is the ratio at time 0 seconds and expressed as a percentage relative to the saturating response, achieved by 25 µM forskolin and 100 µM isobutyl-1-methylxanthine (IBMX).

Real-time cell monitoring

A number of 40,000 HEK293 cells, transfected 24 h before, were seeded in each well of a 96-well E-Plate. The impedance value of each well was automatically monitored for 5 days by the xCELLigence system and expressed as a cell index value. Alternatively, cells were treated the following day after plating with 0.5 mM sodium arsenite (As, Sigma Aldrich) until no cell index was obtained. The background impedance caused by the media was measured using 100 µl in each well prior to seeding of cells and automatically subtracted by the xCELLigence software.

mRNA expression quantitation

RNA from transfected HEK293 cells was isolated with TRIzol (Invitrogen). 5 µg total RNA was treated with DNA-free DNA Removal Kit (Ambion) and used as template for cDNA synthesis with High Capacity cDNA Reverse Transcriptase Kit (Applied Biosystems) according to the manufacturer's instructions. Real-time PCR was performed with predesign probes for PDE10A (Hs01098928_m1, ThermoFisher Scientific) and GAPDH as control gene (Hs02786624_g1) using a TaqMan Gene Expression Assay (ThermoFisher Scientific) on a 7900HT Sequence Detection System (Applied Biosystems) following the manufacturer's instructions. Quantitative analysis was carried out using SDS Version 2.3 software (Applied Biosystems), which calculated the threshold cycle (Ct) values. The relative gene expression of PDE10A was normalized by subtracting the Ct value of the endogenous control from the Ct value of the target assay for each individual well (ΔCt). The fold increase relative to a mock condition with no exogenous PDE10A expression was calculated using $2^{-\Delta\Delta CT}$.

References

1. Wypijewski KJ, et al. (2013) A separate pool of cardiac phospholemman that does not regulate or associate with the sodium pump: multimers of phospholemman in ventricular muscle. *J Biol Chem* 288(19):13808-13820.
2. Rich TC, Tse TE, Rohan JG, Schaack J, & Karpen JW (2001) In vivo assessment of local phosphodiesterase activity using tailored cyclic nucleotide-gated channels as cAMP sensors. *J Gen Physiol* 118(1):63-78.
3. Rich TC, *et al.* (2001) A uniform extracellular stimulus triggers distinct cAMP signals in different compartments of a simple cell. *Proc Natl Acad Sci U S A* 98(23):13049-13054.

Appendix Table S1. Plasmids used in this study

Construct	Source
pcDNA3.1 EPAC1-camps	Prof V Nikolaev, University of Hamburg, Germany
pcDNA3.1 EPAC1-FXYD1	Dr J Day, University of Glasgow, UK
pcDNA3.1 PDE10A1-WT-FLAG	Blue Sky BioServices
pcDNA3.1 PDE10A2-p.A116P-FLAG	Blue Sky BioServices
pcDNA3.1 PDE10A2-p.F300L-FLAG	Blue Sky BioServices
pcDNA3.1 PDE10A2-p.F334L-FLAG	Blue Sky BioServices
pcDNA3.1 PDE10A2-p.Y107C-FLAG	Blue Sky BioServices
pcDNA3.1 PDE10A2-WT-FLAG	Blue Sky BioServices
pCMV GFP	Prof S Moss, Tuft's University, MA
pCMV6 CNGA2 p.C460W/E583M	OriGene Technologies Ltd (MR209871), modified by Genscript
pSV AC3	Prof R Reed, John Hopkins University, MD, USA

Appendix Table S2. **Antibodies employed in this study**

Primary antibody	Supplier	Catalogue number	Host	Dilution	Application
CREB (pS133)	Cell Signalling Technologies	9198	Rabbit	1:1000	WB
Dcp1a	Abcam	ab47811	Rabbit	1:500	IF
E-Cadherin	Cell Signalling Technologies	3195	Rabbit	1:1000	WB
EEA-1	BD Transduction Laboratories	E41120	Mouse	1:250	IF
FLAG	ThermoFisher Scientific	PA1-984B	Rabbit	1:1000<br>1:500	WB<br>IF
FLAG	Sigma-Aldrich	F1804	Mouse	1:500	IF
FLAG	Abcam	ab1257	Goat	1:1000	WB
Flotillin-2	BD Biosciences	610383	Mouse	1:1000	WB
G3BP	Proteintech	13057-2-AP	Rabbit	1:250	IF
GAPDH	Millipore	MAB374	Mouse	1:5000	WB
GM130	BD Biosciences	65120	Mouse	1:250	IF
HDAC6	Abcam	ab1273	Rabbit	1:250	IF
LAMP1	BD Transduction Laboratories	L76620	Mouse	1:100	IF
NSE	Abcam	ab16873	Rabbit	1:1000	WB
PDI	Abcam	ab2792	Mouse	1:500	IF
PDE10A	GeneTex	GTX118886	Rabbit	1:1000	WB
p62	Abcam	ab56416	Mouse	1:1000<br>1:200	WB<br>IF
TIA-1	Proteintech	12133-2-AP	Rabbit	1:250	IF
Ubiquitin	Santa Cruz Biotechnology	sc-8017	Mouse	1:500<br>1:250	WB<br>IF
VASP (pS157)	Cell Signalling Technologies	3111	Rabbit	1:1000	WB
Vimentin-Cy3	Sigma-Aldrich	C9080	Mouse	1:250	IF

Secondary antibody	Supplier	Catalogue number	Host	Dilution	Application
Anti-mouse Al-exa Fluor 488	ThermoFisher Scientific	A32723	Goat	1:500	IF
Anti-rabbit Al-exa Fluor 488	ThermoFisher Scientific	A32731	Goat	1:500	IF
Anti-mouse Al-exa Fluor 594	ThermoFisher Scientific	A11032	Goat	1:500	IF
Anti-rabbit Al-exa Fluor 594	ThermoFisher Scientific	A11037	Goat	1:500	IF
IRDye 680RD Anti-mouse	Li-Cor Bio-sciences	925-68072	Donkey	1:5000	WB
IRDye 800CW Anti-rabbit	Li-Cor Bio-sciences	925-32213	Donkey	1:5000	WB
IRDye 680RD Anti-goat	Li-Cor Bio-sciences	925-68074	Donkey	1:5000	WB

Appendix Figure S1. Distribution and expression of PDE10A variants in different cell lines.

A, Subcellular distribution of the cAMP sensor EPAC-1 used as a cytosolic probe (left) and a modified version with the FYXD1 subunit of Na2+/K+ ATPase in the N-terminal region to translocate the probe to the plasma membrane (right) in transfected HEK293 cells.

B, PDE10A mRNA expression levels in transfected HEK293 cells relative to a mock condition. Log_2-fold change mean values ± SEM is shown and statistical analysis was performed by one-way ANOVA (n = 3).

C, **D**, Immunofluorescence analysis of the recombinant PDE10A-FLAG forms distribution (green) in transfected SH-SY5Y (C) or PC12 cells (D). Scale bar, 10 μm.

E, Immunocytochemical analysis of the different PDE10A2 forms distribution in transfected HEK293 cells after treatment with rolipram (10 uM) for 2 h. Scale bar, 10 μm.

Appendix Figure S2: Analysis of the subcellular localization of PDE10A2 GAF-B mutants.

A, Purification of palmitoylated proteins from HEK293 cells expressing PDE10A1, PDE10A2 WT, F300L or F334L forms.

B, Quantification of palmitoylated PDE10A levels expressed as the mean ± SEM of the palmitoylated/unfractionated ratio relative to PDE10A2 WT. Statistical analysis was performed by one-way ANOVA followed by Dunnett's post hoc test (***p < 0.0001; n = 3).

C, Evaluation of the PDE10A degradation by the lysosome in the soluble fraction of HEK293 cells expressing PDE10A1 or different variants of PDE10A2 and treated with CQ (10 μM) during 12 h.

D, Quantification of PDE10A levels in the soluble fraction after CQ treatment, normalized to GAPDH. Graph represents the mean ± SEM relative to values obtained for the non-treated samples (n = 4).
E, Immunofluorescence showing the distribution of endogenous vimentin (red) in HEK293 cells transfected with the different variants of PDE10A2-FLAG (green) as indicated. The white dashed box shows the selected area for the zoomed images on the right. Scale bar, 10 µm.
F, Immunofluorescence analysis of the colocalization between the PDE10A2-FLAG (green) forms and the cis-Golgi matrix protein GM130 or the ER marker PDI (Protein disulfide isomerase, red). A zoomed area of the PDE10A2 F300L aggregates (indicated as a white dashed box) is shown on the right part of each image as separated channels. Scale bar, 10 µm.

Appendix Figure S3. Characterization of PDE10A2 GAF-B aggregates in HEK293 cells.
A, **B**, **C**, **D**, Immunofluorescence analysis of the colocalization between the PDE10A2-FLAG forms (green) and HDAC6 (A), TIA-1 (B), G3BP (C) or Dcp1a (D, red) in HEK293 cells. Treatment with sodium arsenite (As, 0.5 mM) for 1 h was performed in order to induce production of stress granules. The white dashed box shows the selected area for the zoomed images. Scale bar, 10 µm. Line intensity profiles corresponding to the section shown with dotted line in the image with PDE10A2 F334L condition are located on the right side. PCCs quantification of the indicated PDE10A2-FLAG forms with TIA-1 or G3BP are also shown. Statistical significance was calculated with a two-way ANOVA followed by Bonferroni post hoc test (**$p = 0.0096$, ***$p < 0.0001$; n = 7-15).
E, Proliferation and survival analysis of HEK293 cells expressing PDE10A2 F300L (left panel) or F334L (right panel) compared to a mock condition and cells transfected with the WT variant. Values were obtained using the xCELLigence system and depicted as mean ± SEM (n = 3).

Appendix Figure S4. Characterization of PDE10A2 GAF-B aggregates in rat striatal neurons.
A, Size of PDE10A2 GAF-B aggregates in neurons compared to those observed in different cell lines. Graph represents mean ± SEM and significance was calculated using one-way ANOVA followed by Tukey's post hoc test (***$p < 0.0001$; n =10-50).
B, **C**, **D**, **E**, **F**, **G**, Confocal images showing transfected striatal neurons with the PDE10A2-FLAG forms and immunostained for FLAG (green) and EEA-1 (A), LAMP1 (B), GM130 (C), PDI (D), HDAC6 (E) or Dcp1a (F, red). A white dashed box in the PDE10A2 F334L condition shows the selected area for the zoomed images. Scale bar, 10 µm. Line intensity profiles corresponding to the depicted section with dotted line are located on the right side. PCCs quantification of the indicated PDE10A2-FLAG forms with EEA-1, LAMP1 or Dcp1a are also ilustrated. Statistical analysis was calculated with a one-way ANOVA followed by Dunnett's post hoc test (*$p = 0.0197$; n = 5).

Appendix Figure S1

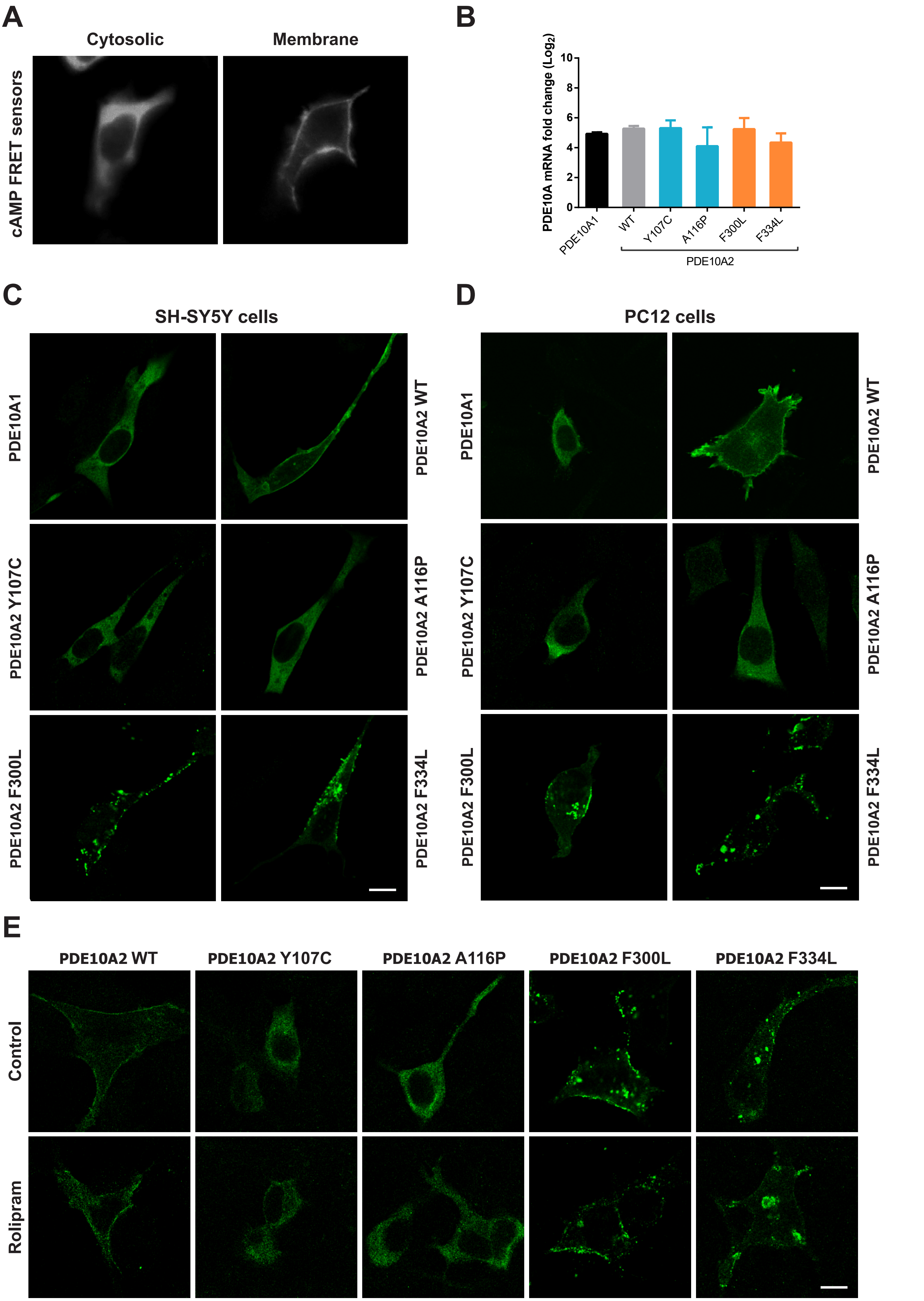

Appendix Figure S2

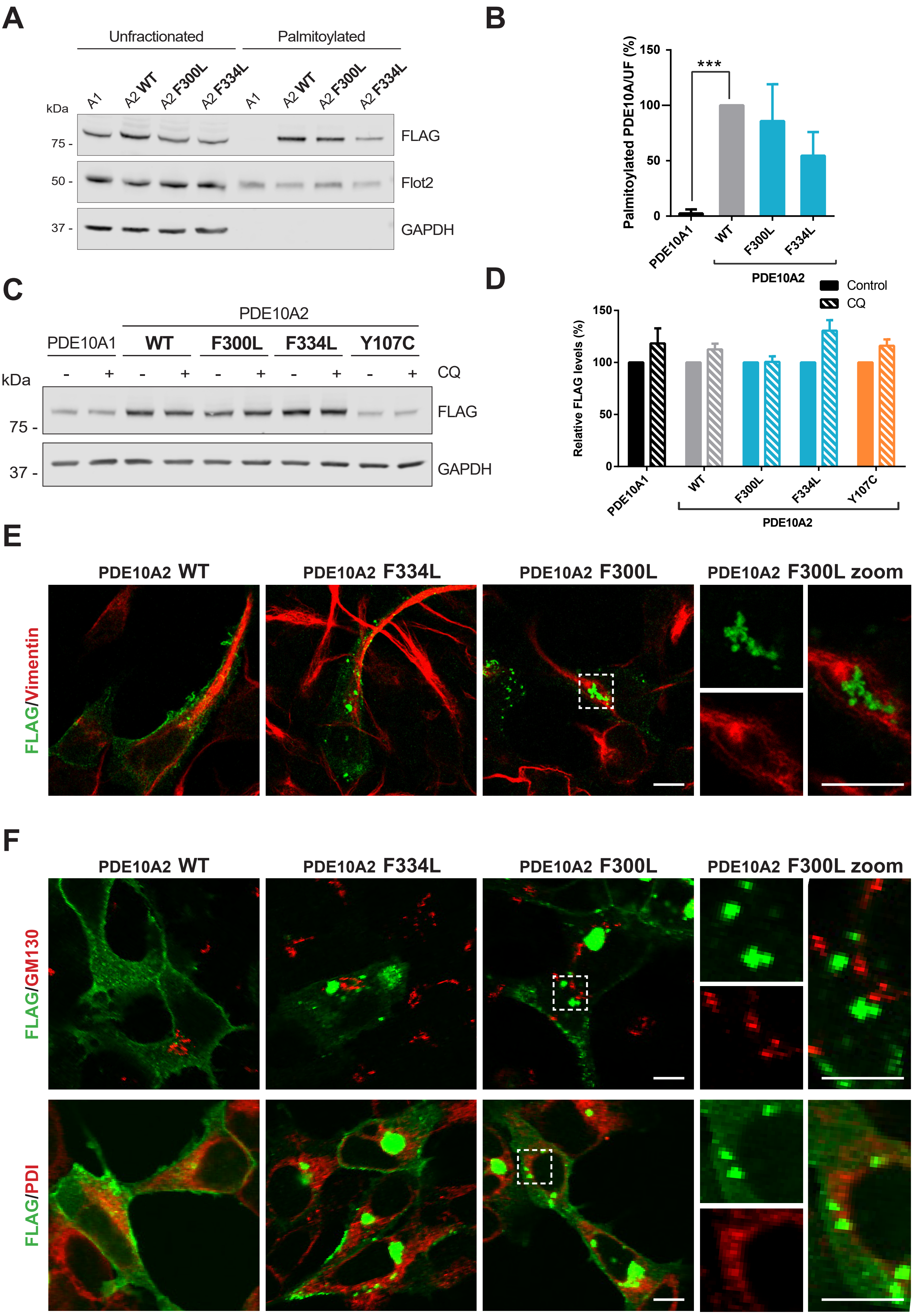

Appendix Figure S3

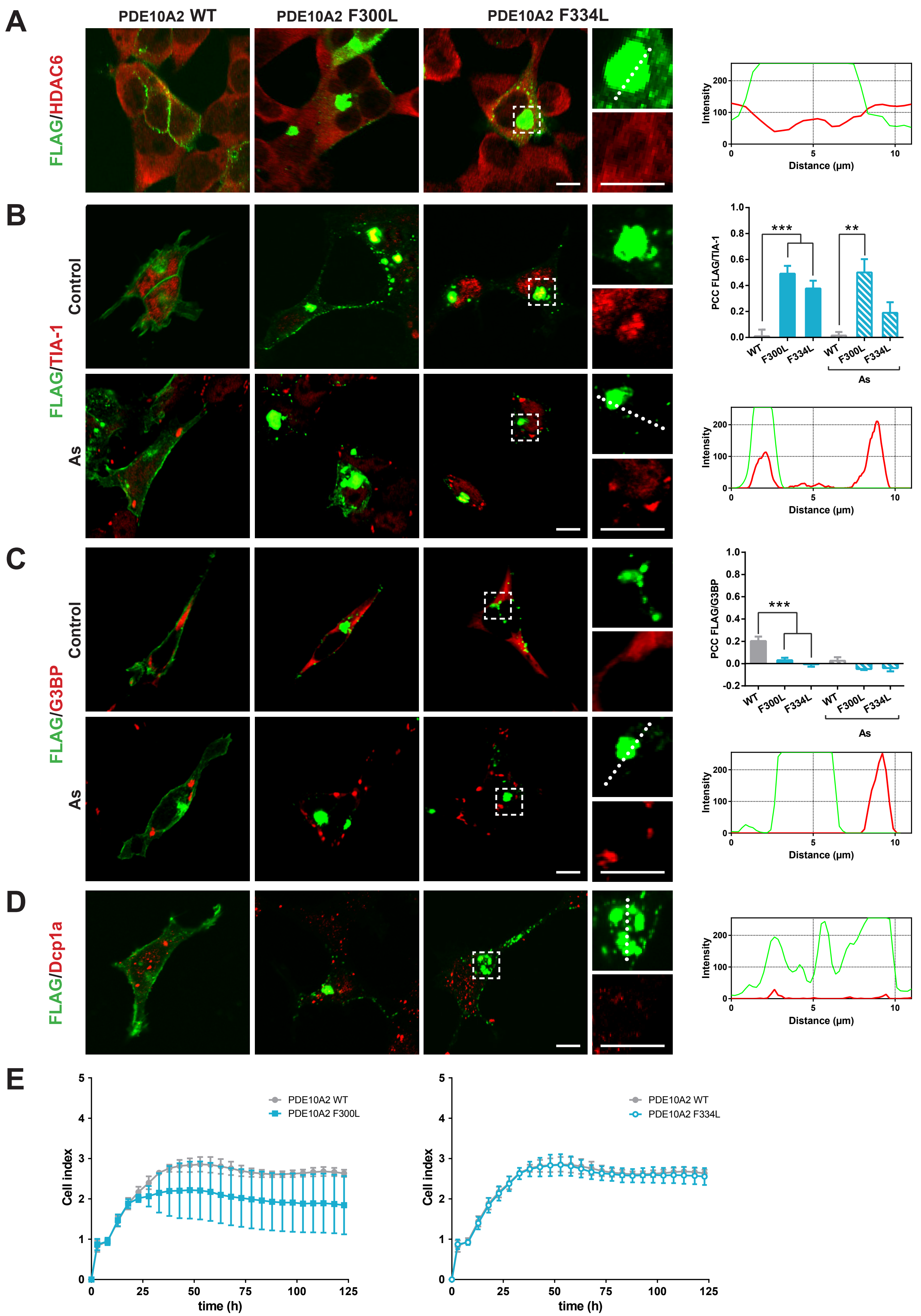

Appendix Figure S4

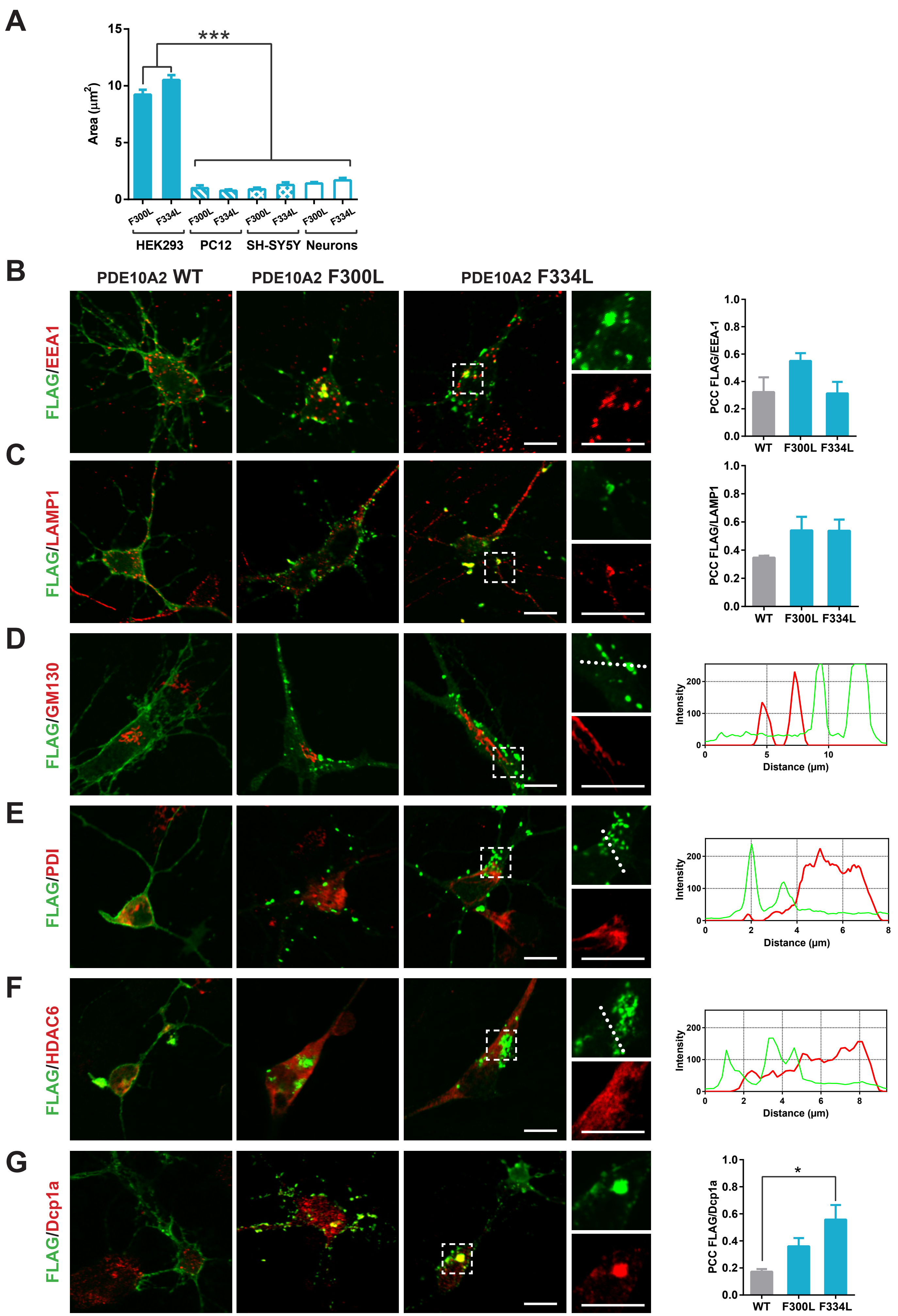

Supplement: Supplementary File [file pnas.1916398117.sapp.pdf]
